# Metabarcoding a diverse arthropod mock community

**DOI:** 10.1111/1755-0998.13008

**Published:** 2019-04-20

**Authors:** Thomas W. A. Braukmann, Natalia V. Ivanova, Sean W. J. Prosser, Vasco Elbrecht, Dirk Steinke, Sujeevan Ratnasingham, Jeremy R. de Waard, Jayme E. Sones, Evgeny V. Zakharov, Paul D. N. Hebert

**Affiliations:** ^1^ Centre for Biodiversity Genomics University of Guelph Guelph Ontario Canada; ^2^ Department of Integrative Biology University of Guelph Guelph Ontario Canada; ^3^ School of Environmental Sciences University of Guelph Guelph Ontario Canada

**Keywords:** community ecology, DNA barcoding, ecological genetics, environmental DNA

## Abstract

Although DNA metabarcoding is an attractive approach for monitoring biodiversity, it is often difficult to detect all the species present in a bulk sample. In particular, sequence recovery for a given species depends on its biomass and mitome copy number as well as the primer set employed for PCR. To examine these variables, we constructed a mock community of terrestrial arthropods comprised of 374 species. We used this community to examine how species recovery was impacted when amplicon pools were constructed in four ways. The first two protocols involved the construction of bulk DNA extracts from different body segments (Bulk Abdomen, Bulk Leg). The other protocols involved the production of DNA extracts from single legs which were then merged prior to PCR (Composite Leg) or PCR‐amplified separately (Single Leg) and then pooled. The amplicons generated by these four treatments were then sequenced on three platforms (Illumina MiSeq, Ion Torrent PGM and Ion Torrent S5). The choice of sequencing platform did not substantially influence species recovery, although the Miseq delivered the highest sequence quality. As expected, species recovery was most efficient from the Single Leg treatment because amplicon abundance varied little among taxa. Among the three treatments where PCR occurred after pooling, the Bulk Abdomen treatment produced a more uniform read abundance than the Bulk Leg or Composite Leg treatment. Primer choice also influenced species recovery and evenness. Our results reveal how variation in protocols can have substantial impacts on perceived diversity unless sequencing coverage is sufficient to reach an asymptote.

## 1 INTRODUCTION

It is generally accepted we have entered a period of unprecedented biodiversity loss (Pimm et al., 2014; Vogel, 2017). Evaluating the scope and regional variation in this loss will require the capacity to quantify shifts in species composition rapidly and on a far larger scale than ever before to better understand and manage ecosystems (Cristescu, 2014; Ji et al., 2013; Moriniere et al., 2016; Waldron et al., 2017). As arthropods account for the majority of terrestrial biodiversity (Medeiros et al., 2013), they are an obvious target for bio‐surveillance. Although they are easily collected in large numbers (Russo, Stehouwer, Heberling, & Shea, 2011), the subsequent processing and identification of specimens has traditionally been a barrier to large‐scale monitoring programmes (Bassett et al., 2012). DNA barcoding, the use of short standardized gene regions to discriminate species, breaks this barrier by enabling nontaxonomists to identify specimens once a reference sequence library is established (Hebert, Cywinska, Ball, & de Waard, 2003; Hebert & Gregory, 2005).

DNA barcode studies initially focused on developing the analytical protocols to construct a specimen‐based reference library (Hebert et al., 2003; Hebert, Penton, Burns, Janzen, & Hallwachs, 2004). Although improved protocols have reduced costs, leading to the analysis of millions of single specimens (Hajibabaei et al., 2005; Hebert et al., 2018; Ivanova, deWaard, & Hebert, 2006), this approach is too expensive to support large‐scale bio‐monitoring programmes. However, by coupling a DNA barcode reference library with the analytical capacity of high‐throughput sequencers (HTS), DNA metabarcoding provides a path to rapid, low‐cost assessments of species composition (Brandon‐Mong et al., 2015; Hajibabaei, Shokralla, Zhou, Singer, & Baird, 2011; Moriniere et al., 2016; Yu et al., 2012). It achieves this goal by generating amplicons internal to the barcode region from bulk DNA extracts which are then sequenced and assigned to operational taxonomic units (OTUs) that are queried against reference sequences to ascertain their source species (see Cristescu, 2014). Studies have now employed this approach to assess species composition in communities of aquatic and terrestrial arthropods (Beng et al., 2016; Elbrecht, Vamos, Meissner, Aroviita, & Leese, 2017; Ji et al., 2013), vertebrates (Sato, Sogo, Doi, & Yamanaka, 2017), diatoms (Vasselon et al., 2017) and fungi (Aas, Davey, & Kauserud, 2017; Bellemain et al., 2012; Tedersoo, Tooming‐Klunderud, & Anslan, 2018). Such metabarcoding analysis routinely reveals more species than morphological approaches while requiring far less time (Brandon‐Mong et al., 2015; Elbrecht, Peinert, & Leese, 2017; Elbrecht, Vamos et al., 2017; Hebert et al., 2018; Ji et al., 2013; Shokralla et al., 2015; Vivien, Lejzerowicz, & Pawlowski, 2016; Yu et al., 2012).

Despite the advantages of metabarcoding, several factors often complicate the recovery of all species in a sample. First, DNA templates derived from the species in a mixed sample are often differentially amplified (Elbrecht & Leese, ; Piñol, Mir, Gomez‐Polo, & Agustí, 2015; Tedersoo et al., 2018). Such bias can arise from either the DNA polymerase (Dabney & Meyer, 2012; Nichols et al., 2018; Pan et al., 2014) or the PCR primers (Clarke, Soubrier, Weyrich, & Cooper, 2014). Polymerase bias involves the differential amplification of templates as a result of variation in their sequence motifs, GC content or length (Dabney & Meyer, 2012; Nichols et al., 2018; Pan et al., 2014). Primer bias arises due to either varying levels of primer mismatch or template degradation (Clarke et al., 2014; Elbrecht & Leese, 2015). The impact of primer mismatches can often be reduced either by lowering annealing temperatures or by raising the degeneracy of the primers (Clarke et al., 2014; Elbrecht & Leese, 2017). However, these “solutions” have a downside; they often increase the amplification of nontarget sequences such as bacterial endosymbionts or mitochondrial pseudogenes, which is especially problematic for eDNA studies (Macher et al., 2018; Smith et al., 2012; Song, Buhay, Whiting, & Crandall, 2008).

The capacity of metabarcoding to recover all species in a bulk sample is further complicated because the component species typically vary by several orders of magnitude in mass and hence in copy numbers of the target template. Unless other factors intervene, this variation in template number means that large‐bodied species are more likely to be recovered (Brandon‐Mong et al., 2015; Elbrecht, Peinert et al., 2017). Because of this effect (in addition to primer bias), efforts to infer species abundance from read counts obtained in metabarcoding studies are at best weak (Elbrecht & Leese, 2015; Piñol et al., 2015). Correction factors can improve such estimates (Thomas, Deagle, Eveson, Harsch, & Trites, 2015; Vasselon et al., 2017), but any method based on the analysis of bulk DNA extracts will fail to accurately determine species abundance.

In addition to factors complicating the recovery of sequences from all species in a bulk sample, sequence variation introduced during PCR, library preparation and sequencing can make it difficult to assign sequences to their source species (Tedersoo et al., 2018). PCR error can be reduced by the use of high‐fidelity polymerases (Lee, Lu, Chang, Loparo, & Xie, 2016; Potapov et al. 2017), but it is more difficult to escape complexities introduced by sequencing error because all second‐generation sequencers have error rates (e.g. 1%–2%) that are high enough to complicate the discrimination of closely related species. Third‐generation platforms, such as Pacific Biosciences Sequel (e.g. Hebert et al., 2018), can produce sequences with much lower error rates, but they currently generate too few reads (~0.3 million/run) to reveal all species in a taxonomically diverse sample (Tedersoo et al., 2018). As a consequence, despite their high error rates, second‐generation platforms (Illumina, Ion Torrent) are commonly used for metabarcoding as they produce many millions of reads per run (Cristescu, 2014; Mardis, 2013). Illumina sequencers generate more reads (20 million–10 billion/run) with lower error rates than Ion Torrent platforms, but the latter instruments can deliver longer reads and can generate results more rapidly (Mardis et al., 2013). It is unclear how severely the choice of HTS platform affects species recovery as their performance has rarely been compared in eukaryotes (Divolli, Brown, Kinne, McCracken, & O’Keefe, 2018). However, work on microbial communities found general agreement between platforms although reads from Ion Torrent platforms were lower quality and more length variable than those from Illumina (Salipante et al., 2014; Tessler et al., 2017). In cases where speed is critical, Ion Torrent platforms have an advantage because of their short run times.

To explore factors affecting the reliability of metabarcoding, we targeted the 658 bp barcode region of the cytochrome *c *oxidase I gene (COI). This gene region has three advantages for metabarcoding studies. First, reference sequences are available for more than 500,000 animal species, far more than any other gene region (Andújar, Arribas, Yu, Vogler, & Emerson, 2018; Porter & Hajibabaei, 2018). Second, because it is protein‐coding, pseudogenes can often be detected because of the presence of frameshift mutations or stop codons. Third, COI has a more rapid rate of evolution than other candidate gene regions, an important advantage in discriminating closely related species, especially given the short amplicons often employed in metabarcoding. Some recent studies have suggested that 16S RNA would be a better target region for metabarcoding studies because of its more conserved priming sites (Deagle, Jarman, Cossiac, Pompanon, & Taberlet, 2014; Elbrecht et al., 2016). This proposal overlooks three disadvantages: (a) it lacks a comprehensive reference database, (b) its slower rate of evolution means that sister species will often share the same sequence (Andújar et al., 2018), and (c) no diagnostic sequence changes are available to recognize pseudogenes. Based on these three weaknesses, it is clear that COI is the better gene region for metabarcoding studies and that effort should be directed towards primer redesign in those cases where current primer sets are ineffective for the group under study (Elbrecht & Leese, 2017).

To examine factors influencing the success in recovering species through metabarcode analysis of COI, we assembled a mock community that included single representatives of 374 insect species. We subsequently used this community to examine the impacts of DNA source, extraction method, PCR protocol, amplicon template and sequencing platform on species recovery. In particular, we examined whether tissue type (abdomens and legs) influences success in the recovery of community composition or whether certain tissues are more prone to false positives. We also wanted to ascertain whether sample processing (bulk vs. individual) affected species recovery. Furthermore, we compared the major HTS platforms to determine whether different sequencing technologies introduced a bias. Specifically, we compared results obtained by analysing read abundance, evenness and species recovery for four amplicon pools on three sequencing platforms (Illumina MiSeq, Ion Torrent PGM, Ion Torrent S5). Two of these amplicon pools derived from the PCR of bulk DNA extracts (abdomen and leg) to test the impact of tissue type. The other two amplicon pools derived from DNA extracts of single legs that were analysed by pooling prior to or after PCR. Finally, we examined species recovery and evenness for two amplicons of differing length on the S5. The overall analytical approach involved evaluation of the relationship between read depth and species recovery for these treatment variables on three sequencing platforms.

## 2 MATERIALS AND METHODS

### 2.1 Assembly of mock community

We began the assembly of a mock community by obtaining COI sequences from 3,044 insects collected in Malaise traps deployed near Cambridge, Ontario, Canada. A DNA extract was prepared from a single leg from each specimen employing a membrane‐based protocol (Ivanova et al., 2006). The 658 bp barcode region of COI was amplified and then Sanger sequenced to link a haplotype to each individual specimen. Amplicons were generated using the primer cocktail of C_LepFolF/C_LepFolR (Hernández‐Triana et al., 2014) with initial denaturation at 94°C for 2 min followed by 5 cycles of denaturation for 40 s at 94°C, annealing for 40 s at 45°C and extension for 1 min at 72°C; then 35 cycles of denaturation for 40 s at 94°C with annealing for 40 s at 51°C and extension for 1 min at 72°C; and a final extension for 5 min at 72°C (Hebert et al., 2018; Ivanova et al., 2006). Unpurified PCR products were diluted 1:4 with ddH_2_O before 2 μl was used as the template for a cycle sequencing reaction (Hebert et al., 2018). All products were sequenced in the forward and reverse directions following standard procedures on an ABI 3730xl DNA Analyzer (Applied Biosystems, Foster City, California, USA).

Because some specimens could not be identified to a species level, we employed the Barcode Index Number (BIN) system which examines patterns of sequence variation at COI to assign each specimen to a persistent species proxy (Ratnasingham & Hebert, 2013). The overall analysis provided sequence records for 803 BINs. From this total, we selected 374 BINs showing > 2% COI sequence divergence from their nearest neighbour under the Kimura 2‐parameter model (Kimura, 1980). The resulting mock community included representatives of 10 orders and 104 insect families. Supporting Information Table S1 lists the taxa in the mock community and provides details on vouchers, their body size (as estimated by abdominal mass) and the GC content of their COI barcode. Following selection of the specimens for inclusion in the mock community, DNA extraction and PCR utilized the protocols described below, and the resultant amplicon pools were analysed on three sequencing platforms.

### 2.2 Experimental design for metabarcode analysis

**Figure 1:**
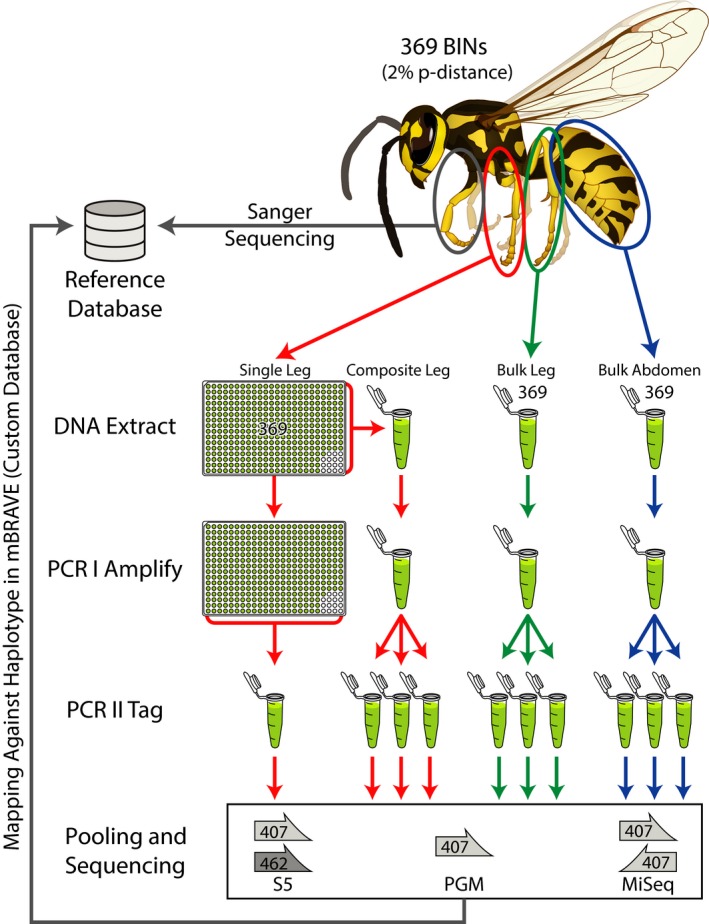
Protocol employed to examine species recovery from the mock community. Four amplicon pools were examined. Two derived from bulk DNA extracts (Bulk Abdomen and Bulk Leg). The others derived from DNA extracts from single legs that were either pooled (Composite Leg) or kept separate (Single Leg) prior to PCR. All four amplicon pools were sequenced on three platforms (Illumina MiSeq, Ion Torrent S5 and Ion Torrent PGM). There were three technical replicates for each treatment except Single Leg [Colour figure can be viewed at http://wileyonlinelibrary.com]

Species recovery was compared for amplicon pools that resulted from four DNA extraction/PCR protocols (Figure 1). Two involved the analysis of amplicons generated from bulk DNA extracts derived from two tissues (Bulk Abdomen and Bulk Leg). The other two treatments involved the initial extraction of DNA from individual legs. The resultant DNA extracts were either pooled prior to PCR to create the Composite Leg treatment or separately amplified and subsequently pooled to create the Single Leg treatment (Figure 1). Although the initial design called for the same specimens to be included in each mock community, this was not possible. The Composite Leg and Single Leg treatments did include the selected array of 374 BINs. However, five of their source specimens either lacked an abdomen or another leg for inclusion in the Bulk Abdomen or Bulk Leg treatments. As a result, five BINs, generally belonging to the same order as the excluded ones, were employed as replacements to maintain 374 BINs per treatment (BOLD:AAA2323, BOLD:AAA2632, BOLD:AAF4234 and BOLD:AAP6354; BOLD:ABV1240). Due to the complexity of our mock community and our desire to evaluate several variables (tissue type, PCR protocol, PCR amplicon and sequencing platform), we did not evaluate multiple biological replicates.

### 2.3 Bulk DNA extractions and PCR

Prior to mock community assembly, the dry abdominal weight of each specimen was measured as a proxy for biomass. DNA extracts for the two bulk samples (Bulk Abdomen and Bulk Leg) were generated with a modified silica membrane‐based protocol (Ivanova et al., 2006). Specifically, the bulk abdomens (combined mass = 1,062.8 mg) and bulk legs (combined mass = 30.9 mg) were lysed overnight in the same relative volume of insect lysis buffer (51.6 ml and 1.5 ml, respectively) with 10 mg/ml of Proteinase K (Invitrogen). Following lysis, a 100‐μl aliquot of each lysate was mixed with 200 μl of binding mix and transferred to an EconoSpin® column (Epoch Life Sciences) before centrifugation at 5,000 g for 2 min. The DNA extracts were then purified with three wash steps. The first wash employed 300 μl of protein wash buffer before centrifugation at 5,000 g for 2 min. Columns were then washed twice with 600 μl of wash buffer before being centrifuged at 5,000 g for 4 min. Columns were transferred to clean tubes and spun dry at 10,000 g for 4 min to remove any residual buffer, then transferred to clean collection tubes and incubated for 30 min at 56°C to dry the membrane. DNA was subsequently eluted by adding 50 μl of 10 mM Tris–HCl pH 8.0 followed by centrifugation at 10,000 g for 5 min. All DNA extracts were normalized to 3 ng/μl prior to PCR. All PCR reactions were composed of 5% trehalose (Fluka Analytical), 1 × Platinum Taq reaction buffer (Invitrogen), 2.5 mM MgCl_2_ (Invitrogen), 0.1 μM of each primer (Integrated DNA Technologies), 50 μM of each dNTP (KAPA Biosystems), 0.15 units of Platinum Taq (Invitrogen), 1 μl of template and HyClone® ultra‐pure water (Thermo Scientific) for a final volume of 6 μl.

### 2.4 Construction of HTS libraries

Two rounds of PCR were used to generate the amplicon libraries destined for sequence characterization on the three platforms. Most first‐round reactions employed a primer cocktail targeting a 407 or 421 bp region of COI, subsequently collectively referred to as the 407 bp amplicon. The 407 bp amplicon was generated using MLepF1 (Hebert et al., 2004) while the 421 bp region was generated by using RonMWASPdegen (Smith et al., 2012) as forward primers; LepR1 (Hebert et al., 2004) and HCO2198 (Folmer, Black, Hoeh, Lutz, & Vrijenhoek, 1994) were used as reverse primers (Supporting Information Table S2). These primers have demonstrated high effectiveness in recovering this segment of the DNA barcode region from diverse lineages of arthropods for Sanger‐based sequencing. They have the advantage of generating an amplicon that is long enough to provide good taxonomic resolution, but short enough to allow characterization on second‐generation HTS platforms. An alternate first‐round PCR targeted a 463 bp amplicon of COI; it was generated with a different forward primer—AncientLepF3 (Prosser, deWaard, Miller, & Hebert, 2016) (Supporting Information Table S2) and sequenced on the S5 platform. Both amplicons employed in this study are longer than those often used in environmental DNA studies, but they have the advantage of maximizing taxonomic resolution and carry no disadvantage when dealing with nondegraded DNA.

All first‐round PCRs were run under the same conditions with initial denaturation of 94°C for 2 min, followed by 20 cycles of denaturation at 94°C for 40 s, annealing at 51°C for 1 min and extension at 72°C for 1 min, with a final extension at 72°C of 5 min. Three technical PCR replicates were generated for three of the treatments—Bulk Abdomen, Bulk Leg and Composite Leg.

Prior to the second PCR, first‐round products were diluted 2 × with dd H_2_O. Fusion primers were used to attach platform‐specific unique molecular identifiers (UMIs) along with sequencing adaptors for Ion Torrent libraries and a flow cell bind for the MiSeq libraries (Supporting Information Table S2). The second PCR was run under the same conditions as the first round for reactions slated for analysis on the Ion Torrent platforms, but the samples for Illumina were amplified following manufacturer's specifications with initial denaturation at 94°C for 2 min, then 20 cycles of denaturation at 94°C for 40 with annealing at 61°C for 1 min and extension at 72°C for 1 min, followed by a final extension at 72°C of 5 min. Supporting Information Table S2 provides all primer sequences and details on sample indexing. For both PCRs, negative controls were used and checked for the lack of a PCR product by visually inspecting an agarose gel.

For each platform, the UMI‐labelled reaction products were pooled prior to sequencing. The two Ion Torrent platforms, the PGM and the S5, differ in their workflows, chemistries and read output. As the S5 is the newer platform, it has a higher read output and generates longer reads (up to 600 bp). The sequence libraries for the S5 were prepared on an Ion Chef™ (Thermo Fisher Scientific) following manufacturer's instructions while those for the PGM were prepared using the Ion PGM™ Hi‐Q™ View OT2 400 Kit and the Ion PGM™ Hi‐Q™ Sequencing Kit (Thermo Fisher Scientific). The PGM libraries were sequenced on a 318 v2 chip while the S5 libraries were sequenced on a 530 chip at the Canadian Centre for DNA Barcoding. Illumina libraries were sequenced (paired end) using the 300 bp reagent kit v3 on an Illumina MiSeq in the Genomics Facility of the Advanced Analysis Centre at the University of Guelph.

### 2.5 Bioinformatics and analysis

All read libraries were uploaded to mBRAVE (Multiplex Barcode Research and Visualization Environment) an online platform for analysing and visualizing metabarcoding data (http://mbrave.net/). Prior to uploading MiSeq runs, read libraries were paired using the QIIME (Caporaso et al., 2010) pair join script (join_paired_ends.py) with a minimum overlap of 20 bp and a maximum difference of 10%. The quality value (QV) of each sequence was evaluated, and all records failing to meet any one of three quality standards were discarded: (a) mean QV < 20; 2) >25% of bp with QV < 20; and 3) >5% of bp with QV < 10. All reads were trimmed to 407 or 463 bp following a 30 bp trim at the 5′ end to remove the forward primer. Reads shorter than 300 bp were discarded for the 407 bp amplicons while a 350 bp threshold was used for the 463 bp amplicon. Retained sequences were viewed as matching a BIN in the custom Sanger library if their distance was < 3% to any reference, a commonly employed threshold (Edgar, 2013). Any reads not matching the Sanger reference library were subsequently queried against four other reference libraries (bacteria, noninsect arthropods, nonarthropod invertebrates and insects). All reads not matching any reference sequence were clustered at an OTU threshold of 2%. Standard analytical parameters were used for all treatments and sequencing platforms. The three replicates for the Bulk Abdomen, Bulk Leg and Composite Leg treatments were also pooled for comparison with the technical replicates.

OTU tables for each run were merged in r v3.4.4 (R Core Team, 2018). To compare BIN accumulation across all samples, we randomly subsampled each run at different read depths for 10,000 replicates using a custom script (Supplemental material). To measure the BIN accumulation for each treatment, we compared the slopes between sequential points at eight read count intervals (10^2^, 10^3^, 10^3.5^ 10^4^, 10^4.5^, 10^5^, 10^5.5^ and 10^6^). Sequential points with a slope of < 0.01 were viewed as indicating that an asymptote had been achieved.

To compare the different treatments and sequencing platforms, we reduced the data set to the 369 shared BINs. Read distributions were visualized using the jamp v0.44 package (https://github.com/VascoElbrecht/JAMP) in r to produce a heat map using the “OTU_heatmap” function. Read distributions across BINs were compared using density graphs generated with ggplot2 v2.2.1 (Wickham, 2009). The relative abundances of all BINs comprising > 0.01% of the overall reads were used to estimate Simpson's index, Pielou's mean evenness and Renyi's entropy implemented in the r package vegan v2.5–1 (Oksanen et al., 2018). Compositional dissimilarity between replicates and treatments was examined using a dendrogram based on the Bray–Curtis index and calculated with vegan. The values for the Bray–Curtis index were also used to generate a nonmetric multidimensional scaling (NMDS) with vegan.

The relationships between read counts and body size, as measured by abdominal mass, and between read count and GC content of the COI amplicon were examined using Kendall Tau correlations in r v3.4.4 (R Core Team, 2018). An analysis of similarity (ANOSIM) with 999 permutations was used to compare species recovery among treatment types, sequencing platforms and between the two amplicons with the r package vegan v2.5–1 (Oksanen et al., 2018). All custom scripts are available as supplementary materials.

The relationship between the read count for each BIN and primer mismatches were investigated for the 407 and 463 bp amplicons. The number of mismatches was quantified by counting the number of nucleotide substitutions between the primer sequence and the template DNA for each BIN. Information on the DNA sequence for the forward primer binding sites was available from the Sanger reads for all 369 BINs. Calculation of mismatches was straightforward for the 463 bp amplicon as it involved a single forward primer. As the 407 bp amplicon was generated with two different forward primers, mismatches were quantified based upon the forward primer with the best match to the template for each BIN. The same two reverse primers were employed to generate the 407 and 463 bp amplicons, but DNA sequence information for template DNA was not available from the Sanger sequence (as it was based on amplicons generated with the same reverse primer). As a result, an alternate reverse primer, C1‐N‐2395d (Simon et al., 1994), was employed to extend each sequence in the 3′ direction, an approach which delivered the desired sequence information for 203 of the 369 BINs. As a consequence, it was possible to examine the relationship between read counts and the number of mismatches between template and forward primer for all 369 BINs and the total mismatch count for the forward and reverse primers for the 203 BINs with template sequences for both regions.

## 3 RESULTS

### 3.1 Run quality

We first compared the output and quality of the reads from the HTS platforms. The S5 and MiSeq generated a similar number of reads (~1 million per replicate), while the PGM generated substantially fewer (~450,000 per replicate). About 60%–65% of the MiSeq reads were filtered during merging of the paired‐end reads, but subsequent filtering was minimal (<1%). The PGM and S5 encountered a similar loss of reads as 45%–50% of the raw reads were filtered (Table 1). The MiSeq reads showed more length consistency and higher quality than those from both Ion Torrent platforms, reflecting their near consistent QV versus the decline towards the 3′ end of the PGM and S5 reads (Supporting Information Figure S1).

**Table 1:**
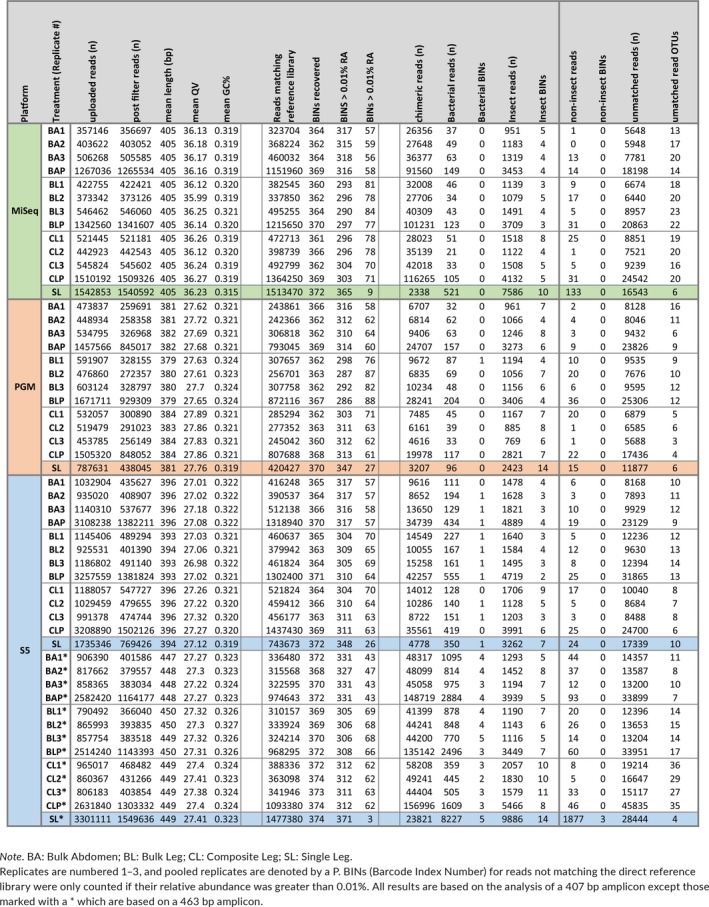
Summary of run results for all treatments. mBRAVE filtering and BIN recovery including false positives are indicated for the four amplicon pools [Colour table can be viewed at http://wileyonlinelibrary.com]

### 3.2 Read depth

**Figure 2:**
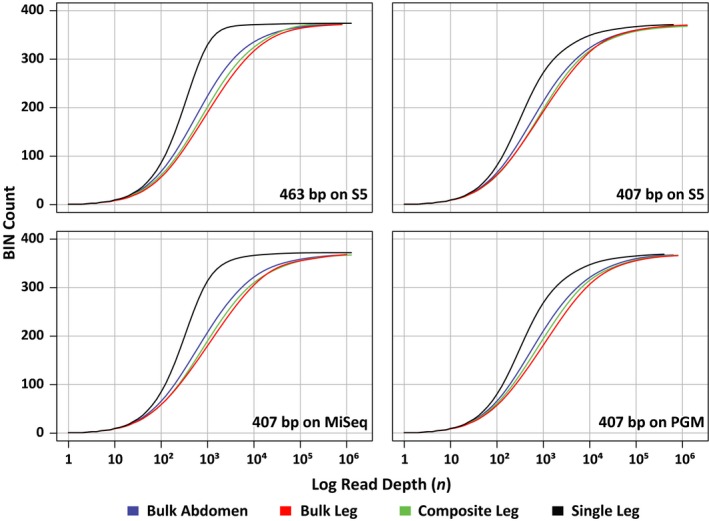
Rarefaction curves showing BIN recovery versus the number of sequences analysed for the four amplicon pools (Bulk Abdomen, Bulk Leg, Composite Leg and Single Leg) on the three sequencing platforms. Two amplicon lengths (407 and 463 bp) were analysed on the S5, but just one (407 bp) on the other platforms [Colour figure can be viewed at http://wileyonlinelibrary.com]

Rarefaction curves were calculated for each of the four treatments and their technical replicates to ascertain if read depths were sufficient to recover all BINs (Figure 2; Supporting Information Figure S2). Although BIN recovery was high in all cases, the Single Leg treatment reached it with far fewer reads of the 407 bp amplicon than the other treatments (10^4–4.5^ vs. 10^4.5–5^ – Supporting Information Table S3). There was evidence of variation among platforms as the PGM needed more reads to achieve an asymptote than the S5 or MiSeq. BIN accumulation curves for the other treatments were similar, but the Bulk Abdomen showed a small, but consistent outperformance versus the Bulk Leg and Composite Leg treatments. The target amplicon also had a substantial impact as just 10^3.5^ reads of the 463 bp amplicon were required for the Single Leg treatment to reach its asymptote (Supporting Information Table S3). The technical replicates showed little divergence on all platforms; they had similar BIN recovery, similar mean read counts per BIN and similar coefficients of variation (Supporting Information Table S1). Pielou's evenness, Simpson's Index, Inverse Simpson's Index, Renyi's diversity and Shannon Indices were also similar across treatments on all platforms (Table 2; Supporting Information Figure S3). Finally, density plots were congruent among technical replicates for all treatments and platforms indicating that different HTS platforms produced similar results (Figure 1; Supporting Information Figure S4).

**Table 2:**
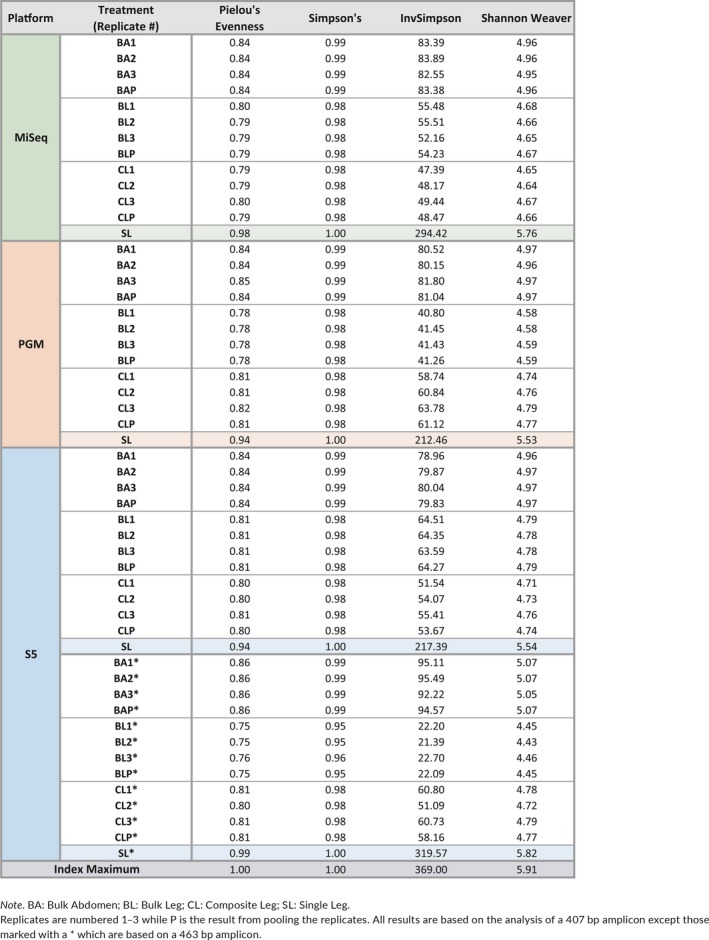
Values for selected diversity indices (Shannon–Weaver, Simpson, Inverse Simpsons and Pielou's Evenness) for the four amplicon pools [Colour table can be viewed at http://wileyonlinelibrary.com]

### 3.3 BIN recovery

**Figure 3:**
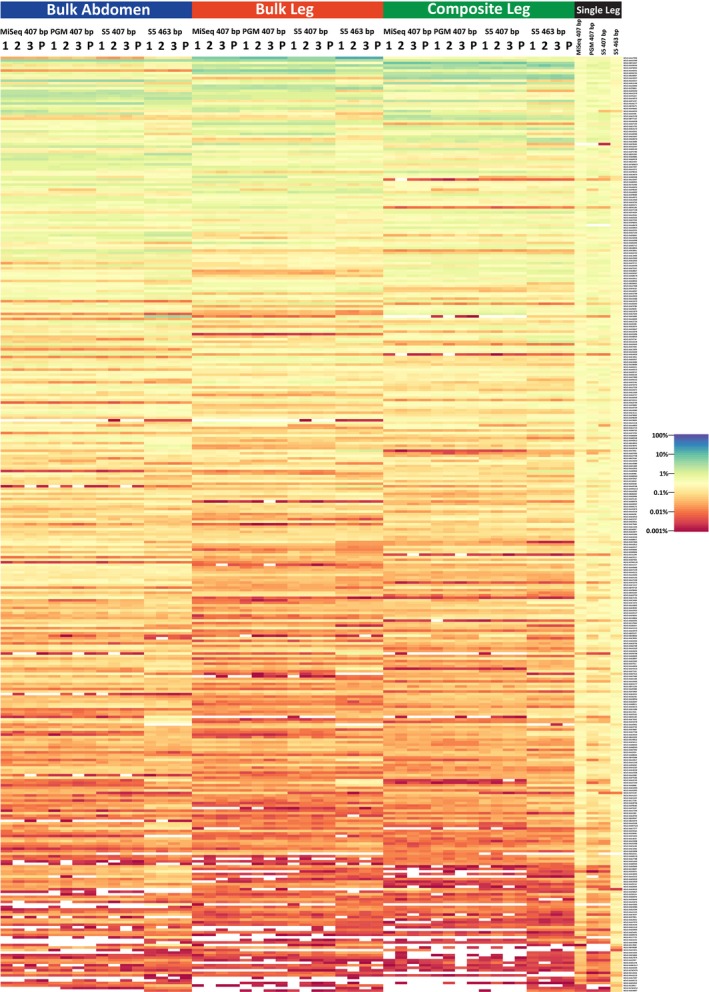
Heat map showing the relative log abundance of the 369 BINs in each treatment for the four amplicon pools. This heat map was created using the JAMP package (https://github.com/VascoElbrecht/JAMP). Technical replicates are indicated with numbers while in silico pooled results are designated by the letter P [Colour figure can be viewed at http://wileyonlinelibrary.com]

When the criterion for BIN recovery was set at one or more reads, all platforms recovered > 98% of the BINs, but only the Single Leg treatment recovered all of them (Figure 3). Differences in recovery success among treatments were greater when the criterion for recovery was set at > 0.01% of the reads. Under this criterion, the Single Leg treatment recovered > 92.5% of the BINs versus 83%–89% for the Bulk Abdomen treatment and 76%–83% for the Composite Leg and Bulk Leg treatments (Table 1). The greater evenness in read count for the Single Leg treatment was striking; it led to lower coefficients of variation, higher diversity indices and Pielou's evenness (Table 2; Figure 3; Supporting Information Figure S3). Density plots of read abundance also demonstrated much higher evenness for the Single Leg treatment, especially for the 407 bp amplicon on the MiSeq and for the 463 bp amplicon on the S5 (Supporting Information Figure S4). These differences were also reflected in BIN recovery, Pielou's evenness and diversity indices (Table 2; Supporting Information Table S2).

### 3.4 BIN abundances

**Figure 4:**
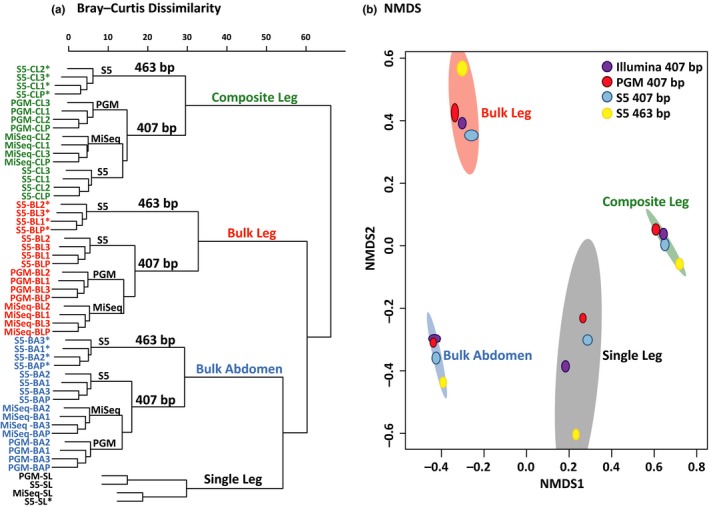
(a) Bray–Curtis dissimilarity dendrogram for the four amplicon pools (BA = Bulk Abdomen, BL = Bulk Leg, CL = Composite Leg and SL = Single Leg). Replicates are numbered 1–3 while P is the result from pooling the replicates. The 463 bp amplicon is indicated with an asterisk (*). (b) Nonmetric multidimensional scaling (NMDS) ordinations using Bray–Curtis dissimilarity for the four amplicon pools. Coloured ellipses represent 95% confidence intervals for the BIN composition of the different treatments using ordiellipse (Oksanen et al. 2012). The shapes within each ellipse represent replicates for the four combinations of sequencing platform–amplicon length for three treatments. No replicates were available for the Single Leg treatment, so it has just four points [Colour figure can be viewed at http://wileyonlinelibrary.com]

Because a single specimen of each BIN was included in the mock community, the proportion of sequences from each should, in the absence of bias, be similar across sequencing platforms, amplicons and treatments. In practice, the relative abundances of the BINs varied markedly. Perceived abundance of the 369 taxa based on their read counts varied more than 11,000‐fold for the Bulk Abdomen, Bulk Leg and Composite Leg treatments, and 4,000‐fold for the Single Legs. A single‐link dendrogram based on Bray–Curtis dissimilarity values indicated that samples clustered first by treatment, next by amplicon length and finally by sequencing platform (Figure 4a). An analysis of similarity using Bray–Curtis distances affirmed significant differences in BIN abundances by treatment type (*p* = 0.001, *R* = 1), amplicon length (*p* = 0.027, *R* = 0.17), but not by sequencing platform (*p* = 0.13, *R* = 0.037) (Figure 4b; Supporting Information Figure S5).

### 3.5 Primer mismatches and read count

Examination of the relationship between the read count for each of the 369 BINs and its number of mismatches from the forward primer revealed a strong negative relationship. BINs with a high mismatch count were typically represented by few reads. For example, very few reads were recovered from the only BIN in the order Dermaptera and this was associated with a high mismatch Index from the forward primers for both the 407 and 463 bp amplicons. Considering all taxa, BIN recovery was substantially higher for the 463 bp amplicon than for the 407 bp amplicon (Table 2; Supporting Information Figure S3) reflecting the fact that its forward primer better matched the template DNA (18 BINs had > 3 mismatches) versus the forward primer for the 407 bp amplicon (62 BINs with > 3 mismatches) (Supporting Information Tables S1 and S4). The impact of these mismatches was clear; read count and relative abundance of BINs declined after two mismatches for the Bulk Abdomen, Bulk Leg and Composite Leg treatments and after four mismatches for the Single Leg. Examination of the joint impact of forward and reverse primer mismatches for 203 BINs similarly showed a significant decline in read count and relative abundance after four mismatches for the Bulk Abdomen, Bulk Leg and Composite Leg treatments and after seven mismatches for the Single Leg (Supporting Information Figure S6). Kruskal–Wallis tests showed that read depth declined significantly with an increasing number of primer mismatches for the forward primers for both the 407 and 463 bp amplicons (*p* < 0.0001) and for the summed primer mismatches (5′ + 3′) for the subset of 203 BINs (*p* < 0.0001).

### 3.6 Impacts of biomass and nucleotide composition on read count

Other factors also explained some of the variation in read counts among BINs. There was, for example, a weak negative correlation (*r* ^2^ < 0.10) between the GC content of an amplicon and its read count, excepting the Single Leg treatment on the MiSeq where it was higher (*r* ^2^ = 0.32) (Supporting Information Figure S7). A weak positive correlation (*r* ^2^ = 0.24–0.28) was also apparent between the abdominal mass of a BIN and its read count on all platforms (Supporting Information Figure S8).

### 3.7 Nontarget sequences

Each run recovered some sequences with substantial sequence divergence from the Sanger reference library (Table 1). The incidence of nontarget sequences for the 407 bp amplicon was slightly lower (4%–6%) on the PGM and S5 platforms than on the MiSeq (8%–10%). Interestingly, the 463 bp amplicon had substantially more nontarget reads (15%–17%). After excluding the relatively few chimeras (1.5%–12.5%), half the reads from the nontarget sequences failed to match any sequence in the supplemental libraries. Of those that did find a match, most were arthropods.

### 3.8 Taxonomic bias

There was evidence of differing taxonomic bias in the read counts for BINs between the two amplicons. For example, Orthoptera, Lepidoptera and Diptera dominated the 407 bp sequences from the Bulk Abdomen and Bulk Leg treatments while Lepidoptera, Mecoptera, Diptera and Coleoptera dominated those for the 463 bp amplicon (Supporting Information Table S5). The 463 bp amplicon also showed more variation among treatments than the 407 bp amplicon (Supporting Information Table S5). Among the bulk samples, relative abundance differed among treatments. For example, the relative abundance of Lepidoptera and Mecoptera was lower, while Diptera and Orthoptera were higher in the Composite Leg than in the Bulk Leg and Bulk Abdomen treatments. The proportion of read counts for Trichoptera showed particularly large variation, being 5–25X higher for the Bulk Leg than the Bulk Abdomen and Composite Leg treatments across all platforms and for both amplicons.

## 4 DISCUSSION

Metabarcoding is a powerful tool for characterizing biodiversity patterns (Cristescu, 2014), but data interpretation is complicated by several factors. PCR amplification bias and variation in the copy number of template DNA from the source specimens not only make it impossible to estimate abundances, but can impede the recovery of all species (Beng et al., 2016; Elbrecht & Leese, 2015; Ji et al., 2013; Yu et al., 2012). Although prior studies have revealed these complexities, there has been limited evaluation of the strength of their influence on interpretations of taxon diversity. To address this gap, the present study examined the impact of diverse factors including source DNA, PCR primers, sequencing platform and sequencing depth on species recovery from a diverse assemblage of insects. Both primer sets demonstrated their effectiveness for metabarcoding as they recovered > 98% of the 374 species in a taxonomically diverse mock community (10 orders, 104 families). However, it did require substantial sequence coverage to recover these species because of their varied amplicon abundance. Variation in body size of the species and shifts in the GC composition of their COI templates partially accounted for the divergence in amplicon abundance, but the mismatch count between primers and template DNA had a greater effect. While new primer sets can be designed to reduce such mismatches, it will never be possible to entirely escape them in any large assemblage of phylogenetically diverse species.

### 4.1 Sequencing depth

Because of diversity in amplicon abundance among taxa, sequencing depth has a strong impact on taxon recovery and hence perceived diversity patterns (Leray & Knowlton, 2015; Leray & Knowlton, 2017). Species with low representation in the amplicon pool, because of primer–template mismatches or low template concentrations linked to rarity or small body size, are likely to be missed unless sequencing depth is very high. When species are overlooked, alpha diversity is underestimated, and beta diversity is exaggerated (Bellemain et al., 2012; Sickle et al., 2015; Sickle et al., 2015; Yamamoto et al., 2017). There is a simple way to assess whether sequencing effort has been adequate; the slope of the rarefaction curve is zero when all species have been recovered (Lanzen, Lejang, Jonassen, Thompson, & Troedsson, 2017). Technical replicates are also useful because every replicate should include the same OTUs when sequence coverage is adequate. Although taxon richness was fixed in our study, we employed a slope for the rarefaction curve of < 0.01 as the criterion to decide whether taxon diversity had achieved an asymptote. This criterion has the advantage of being applicable in situations where the species count is unknown, as is regularly the case in nature. Based upon this criterion, we detected up to 100‐fold differences in the level of sequencing that was required to achieve an asymptote among the four treatments and two amplicons examined in this study.

BIN accumulation curves indicated that the read depth employed in this study allowed all four treatments to meet a slope of < 0.01. However, the Single Leg treatment reached this value with much lower read depth than the bulk samples due to its relative protection from the impacts of PCR bias (Nichols et al. 2018; Pan et al., 2014, Dabney & Meyer, 2012; Elbrecht & Leese, 2015). Interestingly, the other three treatments showed similar BIN accumulation curves on all three sequencers, suggesting shared factors constrain BIN recovery.

### 4.2 Sequencing platforms

The three sequencing platforms generated similar estimates of BIN diversity. However, results from the MiSeq had advantages over those from the PGM and S5 for both clustering algorithms and for haplotype analysis (Elbrecht, Varnos, Steinke, & Leese, 2018) reflecting its delivery of full‐length, higher fidelity reads. In particular, the paired‐end protocol consistently recovered sequences for the full 407 bp amplicon, while those from the PGM and S5 were often truncated and possessed more indels. Finally, the MiSeq reads had consistently higher mean QV. Because these factors simplified data analysis (Edgar et al., 2013; Mardis et al., 2013) and sequencing costs were similar, the MiSeq is currently the best platform for metabarcoding (Mardis et al., 2013).

### 4.3 Impacts of analytical protocols

Our four treatments made it possible to compare the impact of targeting different tissues, employing different DNA extraction regimes and using different PCR protocols. Despite their similar tissue input and DNA extraction regime, the Single Leg treatment achieved asymptotic diversity much more rapidly than the Composite Leg treatment, indicating how separate PCR reactions reduce amplification bias. By contrast, BIN accumulation curves and diversity indices for the Composite Leg treatment were similar to those for the Bulk Leg and Bulk Abdomen, indicating that DNA extraction was equally effective whether carried out on single specimens or on bulk samples (Table 2). The comparison of the results for the bulk/composite samples did reveal more nuanced differences as the number of reads for particular taxa varied among these three treatments despite similar BIN recovery profiles (Figure 3). These differences likely stem from differential leg/abdomen mass ratios among species which led to varied mitochondrial copy numbers for the component species among treatments. Certainly, mitochondrial copy number varies among tissues and among species (Cole, 2016; Veltri, Espiritu, & Singh, 1990). Future efforts to explore this relationship and its importance to metabarcoding studies should quantify copy number differences between tissues and species. In the absence of such information, copy number bias due to biomass or species differences can be reduced by partitioning the specimens in a bulk sample into size fractions (Elbrecht, Peinert et al., 2017; Vivien et al., 2016).

Variation in read counts for the taxa in any bulk sample is, as already noted, strongly influenced by primer–template mismatches. Although degenerate primers (Elbrecht & Leese, 2017; Moriniere et al., 2016; Yu et al., 2012) and improved primer sets (Clarke et al., 2014; Elbrecht & Leese, 2017; Leray & Knowlton, 2017) can reduce such bias, it cannot be avoided unless all target species possess identical sequences for the primer binding sites, a condition that will never be satisfied for a large assemblage. However, efforts to target highly conserved regions can improve the situation. For example, the BIN accumulation curve for the 463 bp amplicon reached its asymptote with much lower read coverage than for the 407 bp. Further effort to develop primers that maximize primer–template matches for diverse taxa will reduce the sequencing effort needed to recover all taxa. So too will strategies that minimize mismatches by partitioning bulk samples into major taxonomic groups (Bellemaine et al., 2012; Cristescu, 2014; Moriniere et al., 2016; Tedersoo et al., 2015). Lowering the variation in recovery success linked to size differences among species can be achieved by partitioning the species present in bulk samples into subsets with similar size (Elbrecht, Peinert et al., 2017; Moriniere et al., 2016; Vivien et al., 2016). Currently, the only means to fully escape the varied factors influencing sequence recovery is to process specimens individually through the entire analytical chain from DNA extraction to sequencing but this approach is too costly for large biodiversity surveys (Ji et al., 2013).

### 4.4 BIN recovery

Although sequences were recovered from most of the BINs in each treatment, this outcome shifted when recovery success was defined as only those BINs comprising > 0.01% of the read count, a criterion often employed to exclude low‐frequency sequences that are chimeras, contaminants or sequencing errors (Leray & Knowlton, 2017). Under this criterion, BIN recovery was substantially higher (>92.5%) for the Single Leg treatment than for the other three (76%–89%). Interestingly, the Bulk Abdomen treatment showed higher BIN recovery than the Bulk Leg and Composite Leg treatments, perhaps reflecting more similar mitochondrial copy numbers among abdomens than legs (Figure 3) (Cole, 2016; Veltri et al., 1990). As expected, BIN recovery was more efficient for the 463 bp than the 407 bp amplicon because of its higher primer–template correspondence. There was also less taxonomic bias (Table 2) for the 463 bp than for 407 bp amplicon in three treatments (Bulk Abdomen, Composite Leg and Single Leg).

### 4.5 False positives, negatives and unmatched OTUs

Although most BINs were recovered in each treatment, some comprised < 0.01% of the counts, creating false negatives that would underestimate alpha diversity. As in other metabarcoding studies (Vivien et al., 2016; Ficetola et al., 2015; Brandon‐Mong et al., 2015; Port et al., 2016), false positives were also encountered, likely reflecting eDNA associated with specimens or contamination during sample processing (Port et al. 2016) or NUMTs (Song et al., 2008). Their impact can be reduced by employing curated reference libraries to discriminate sequences that derive from known species versus those that represent pseudogenes (Bergsten et al., 2014; Braukmann, Kuzmina, Sills, Zakharov, & Hebert, 2017; Hebert et al., 2003; Landi et al., 2014; Zimmerman et al., 2014). As well, negative controls make it possible to identify reads that derive from contamination events during sample processing (Port et al. 2016).

Although we expected the Bulk Abdomen treatment to generate more nontarget sequences than the others, reflecting template DNA from the digestive tract, this was not the case. In retrospect, it seems likely that DNA molecules from this source were too degraded to be recovered via the 407 bp and 463 bp amplicons. Certainly, most metabarcoding studies on gut contents or faecal samples have employed shorter amplicons (Hajibabaei, Spall, Shokralla, & Konynenburg, 2012; Kartzinel et al. 2015; Linard, Arribas, Andújar, Crampton‐Platt, & Vogler, 2016). Because legs have a much higher surface area to volume ratio than abdomens, they may bind more eDNA, leading to their slightly higher recovery of nontarget DNA.

Approximately 1%–4% of the filtered reads did not match any sequence in the reference library. This varied slightly by platform with the MiSeq showing fewer unmatched reads (1.07%–1.73%) than the S5 (1.64%–2.50%) and PGM (2.06%–3.11%). Similarly, the 407 bp amplicon on the S5 had fewer nonmatching reads (1.64%–2.50%) than the 463 bp amplicon (1.84%–4.10%) on this platform. Some of the unmatched reads are undoubtedly derived from NUMTs, PCR errors and sequencing errors. Although we did not evaluate the incidence of pseudogenes, they likely represent some of the highly divergent unidentified OTUs (Leray et al., 2015). Their frequency among terrestrial arthropods needs to be further explored through specimen‐based analysis. The Miseq produced the highest quality reads, suggesting that the higher incidence of unmatched reads on the Ion Torrent platforms reflect, in part, sequencing errors, especially the PGM which has a steep decline in quality towards the 3’ end (Supporting Information Figure S1). PCR errors introduced by the DNA polymerase can be exacerbated by sequencing errors (Dabney & Meyer, 2012; Nichols et al., 2018; Pan et al., 2014). Because long amplicons improve taxonomic resolution, their use should be standard for metabarcoding studies unless template DNA is degraded.

## 5 FUTURE METHODS AND CONCLUSIONS

This study has established that current PCR‐based protocols for metabarcoding can recover most species in a diverse assemblage of insects when sequencing depth is adequate. Specifically, it required from 100,000 to 500,000 reads (300x–1,000x average read depth) to recover > 95% of the 374 species from bulk DNA extracts. Although the choice of sequencing platform had little impact on final results, the higher quality of sequences generated by Illumina MiSeq simplified data analysis. Given current analytical costs (Supporting Information Table S6), there is a clear justification to search for protocols that make it possible to reveal the species in any assemblage with limited sequencing effort. The importance of employing primer sets that minimize mismatches with template DNA was evidenced in this study by the fact that a tenth as many sequences were required to recover 95% of the species for the 463 bp than the 407 bp amplicon. Given such impacts, further effort to design primers which minimize mismatches for DNA extracts from diverse taxonomic assemblages (e.g. zooplankton, insects) are important. Success should be facilitated because 3rd generation sequencers can analyse longer amplicons, permitting the use of primer sets that target regions of COI where sequences are constrained because they code for amino acids that bind substrates or cofactors. However, because sequence variation is inevitable in any diverse taxonomic assemblage, the copy number of sequences in the amplicon pool will diverge from their abundance in the original DNA extract.

To escape such bias, PCR‐free protocols have been proposed (Liu et al., 2016, Tang et al., 2014). In their simplest implementation, they involve the random analysis of genomic fragments, followed by the exclusion of reads that fail to match a sequence in the reference library. This approach means that many sequences do not contribute to a species assignment. For example, if analysis focused on COI‐5’, approximately 1 in every 2,000 sequences would be retained because it represents just 5% of the mitochondrial genome and mitochondrial DNA represents just 1% of the total DNA in a cell. Hence, to recover a single barcode sequence from 374 species, presuming an identical number of COI templates for each taxon and sampling one copy per species, 750,000 sequences would be needed. In practice, far more sequences would be required to overcome the impacts of random sampling and body size. To recover 95% of the species in the mix, assuming random sampling and equal template count for each species would require an average of ~3 sequences per species, based on $N\sum_{i=0}^{k}\frac{1}{N-i-1}$ , a variation of the coupon‐collector's problem (Motwani & Raghavan, 1995) where *N* is the number of species and k is the recovered subset, raising the required number of sequences to 2.25 million. Further sequencing would be needed to compensate for the variation in template numbers linked to body size variation. The species of arthropods examined in this study varied 7,500‐fold in body mass. Assuming that mitochondrial copy number and metabolic rate scale in a similar fashion with body mass^0.66^ (Burgess et al., 2017), the largest species examined in this study should have possessed about 360 times more target template than the smallest species. Given of this difference, some 810 million sequences would be needed to recover 95% of the species in the assemblage. More conservatively, if specimens with the lowest 5% body mass are excluded, there is a 178‐fold difference between the smallest and largest species. The largest species will have 30 times more target template, requiring 67.5 million reads to recover the species in the assemblage. Since the simplest implementation of PCR‐free approaches is so inefficient, more advanced protocols employ baits to enrich for the target gene region (Dowle, Pochon, C. Banks, Shearer, & Wood, 2015), but they can create interpretational complexities when capture efficiencies vary among taxonomic groups.

It is important to emphasize that no current metabarcoding protocol allows the estimation of species abundances. PCR‐based methods fail because of distortions in the amplicon pool introduced by variation in primer binding. PCR‐free methods can reveal the abundance of each template in the total DNA extract, but this count of COI molecules cannot predict species abundance because a high value might derive from a few adults or many juveniles. Given these barriers to abundance assessment through metabarcoding, it is worth noting that specimen‐based analysis can deliver this information with very limited sequencing effort. For example, just 374 Sanger reads or 3,000 reads of a UMI‐tagged DNA pool on the Sequel platform (Hebert et al., 2018) would have revealed the presence and equal abundance of each species in the current sample. Although neither PCR‐based nor PCR‐free metabarcoding can deliver accurate information on species abundance, the two approaches can deliver complementary information on species composition. Because the amplicon pool generated by PCR is influenced by variation in primer binding, the impact of variation in body size is diminished since small species whose COI template closely matches primer sequences will be well represented in the amplicon pool. By contrast, PCR‐free methods can aid the recovery of large species that can be overlooked in PCR‐based studies because of poor amplification.

In summary, this study has established that PCR‐based metabarcoding provides a cost‐effective way to recover information on the species composition of insect communities because current COI primer sets are broadly effective, and a well‐provisioned reference library is available. The optimal solution may be different for other taxonomic groups, especially those where primers fail to amplify many species or where amplification bias is extreme. However, in such cases, it remains important to try to overcome these barriers rather than simply capitulating. It is also worth emphasizing that many natural communities possess greater complexity than the assemblage examined in this study; they include more species and the abundances of these species show great variation. Given these complications, community characterization through metabarcoding will often require both intensive sequencing and improved informatics support to recognize sequences that reflect rare species rather than analytical artefacts.

## AUTHOR CONTRIBUTIONS

The study was conceived and designed by PDNH, EZ, TWAB, DS, SR, JRD, NI, and SP; The research was performed by NI, JS, SP, and TWAB; The analysis of data was performed by TWAB and VE; Analytical tools were contributed by SR and VE; The manuscript was written by TWAB and PDNH with input and revisions from DS, SR, VE, EZ, NI, SP, and JS.

## Data Availability

Further details on the treatments are available at the following DOIs: Bulk Abdomen and Bulk Leg: https://doi.org/10.5883/DS-NGS375A; Composite Leg and Single Leg: https://doi.org/10.5883/DS-NGS375B. All raw HTS sequence data is available in NCBI's Short Read Archive (SRP158933).
