# Supplementary material for: Metabarcoding a diverse arthropod mock community
Source: Mol Ecol Resour. 2019 Apr 20;19(3):711–27. doi: 10.1111/1755-0998.13008 (PMC6850013; doi:10.1111/1755-0998.13008)
Supplement: Supplementary file 1 [file MEN-19-711-s001.zip › men13008-sup-0008-FigS8.pdf]

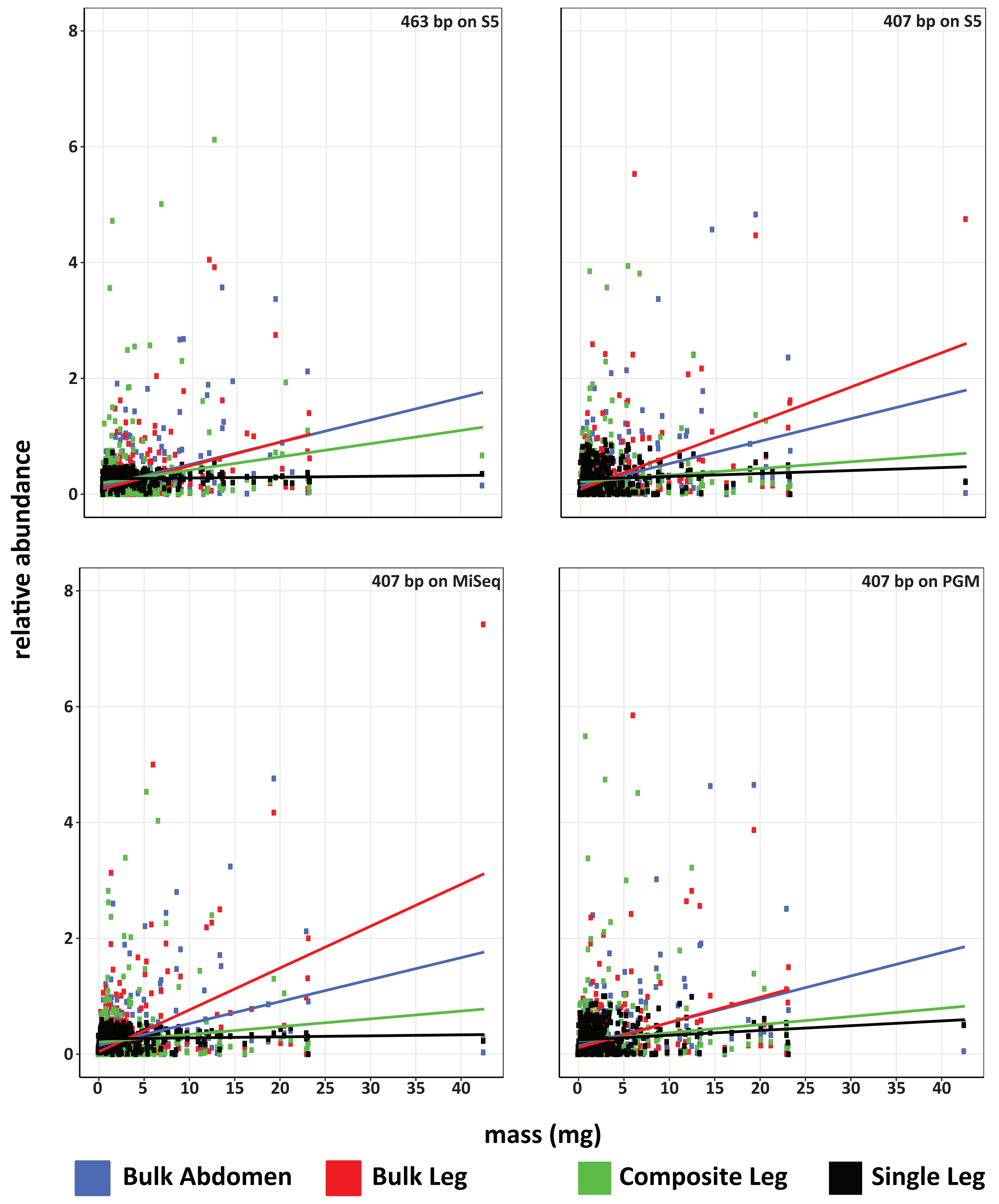

463 bp on S5
407 bp on S5
407 bp on MiSeq
407 bp on PGM
relative abundance
mass (mg)
0
2
4
6
8
0
5
10
15
20
25
30
35
40
Bulk Abdomen
Bulk Leg
Composite Leg
Single Leg
