# Supplementary figures and images for: Metabarcoding a diverse arthropod mock community
Source: Mol Ecol Resour. 2019 Apr 20;19(3):711–27. doi: 10.1111/1755-0998.13008 (PMC6850013; doi:10.1111/1755-0998.13008)

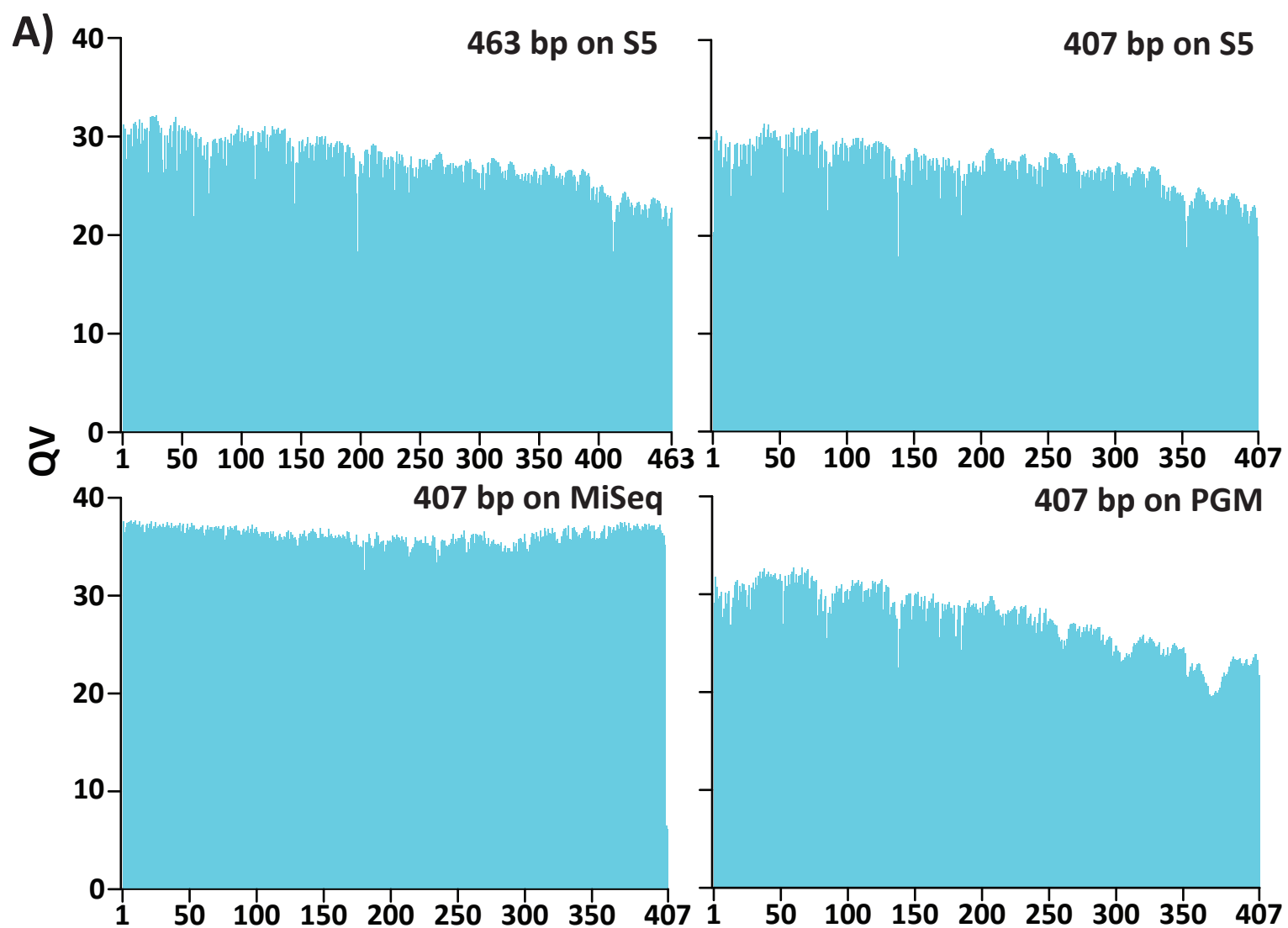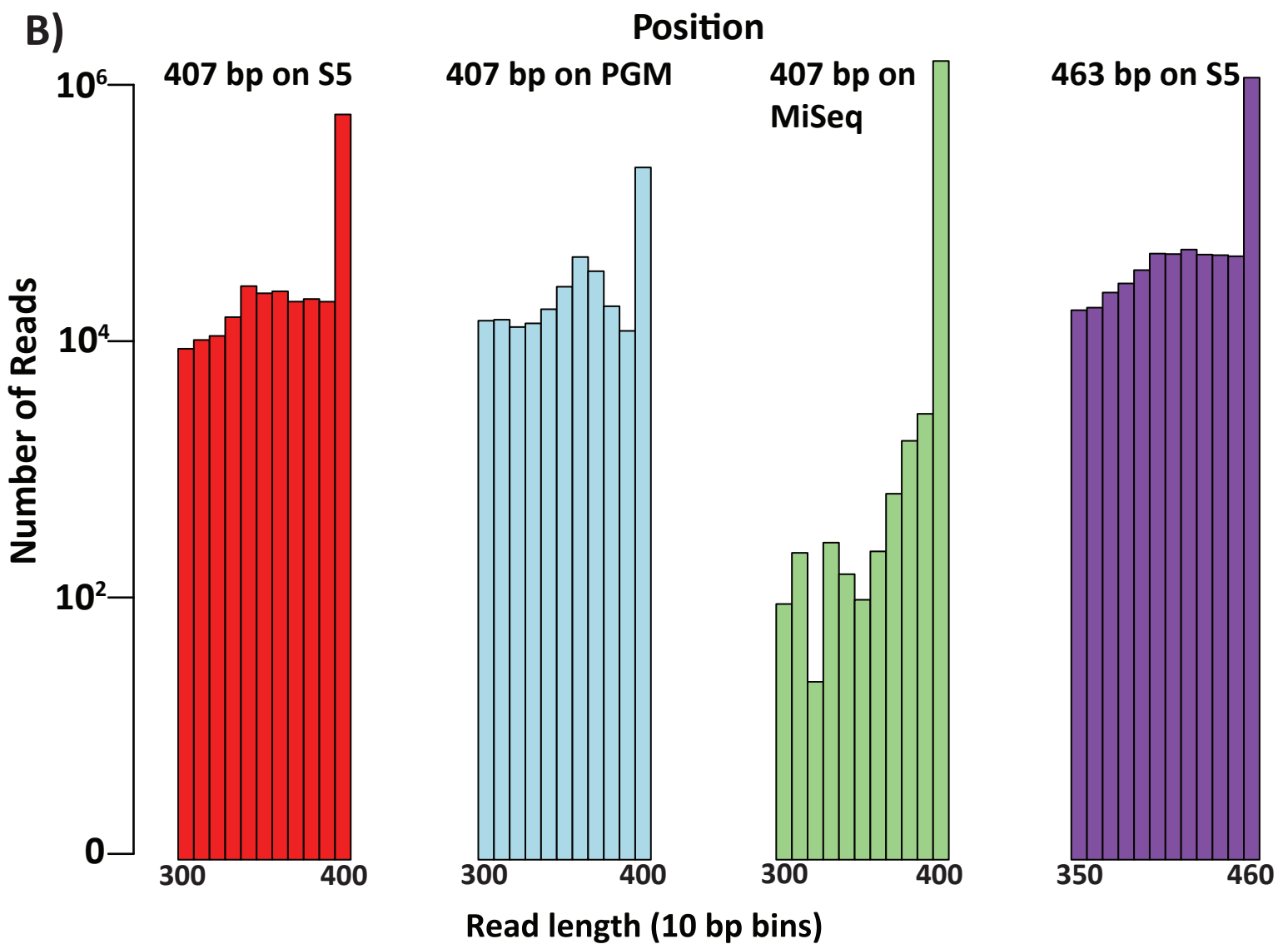

Supplement: Supplementary file 1 [file MEN-19-711-s001.zip › men13008-sup-0001-FigS1.pdf]

**BIN Count**

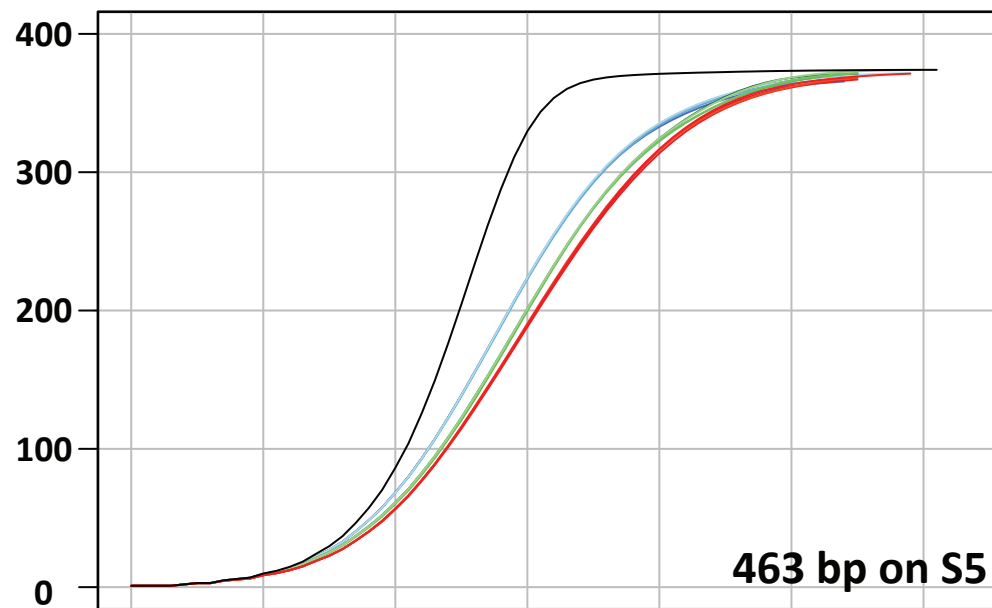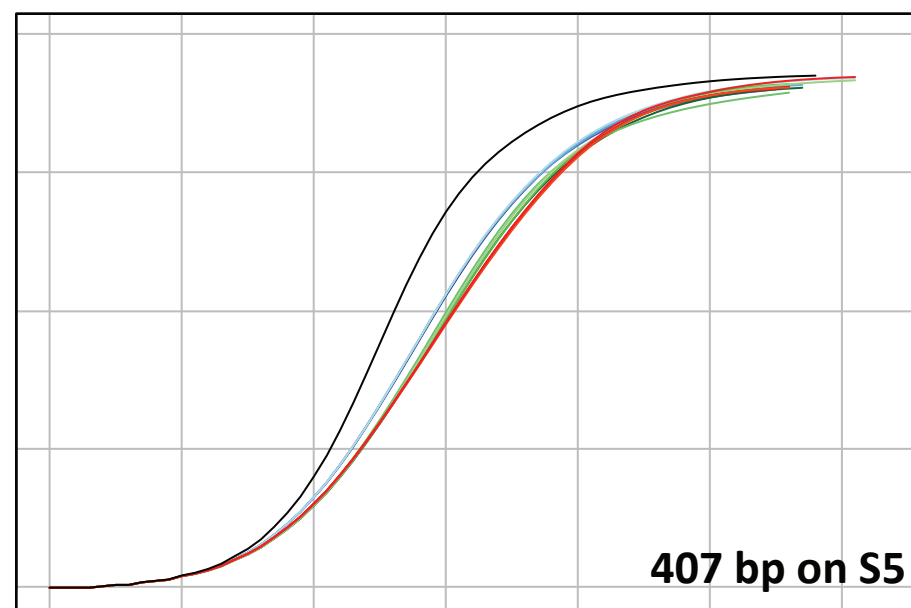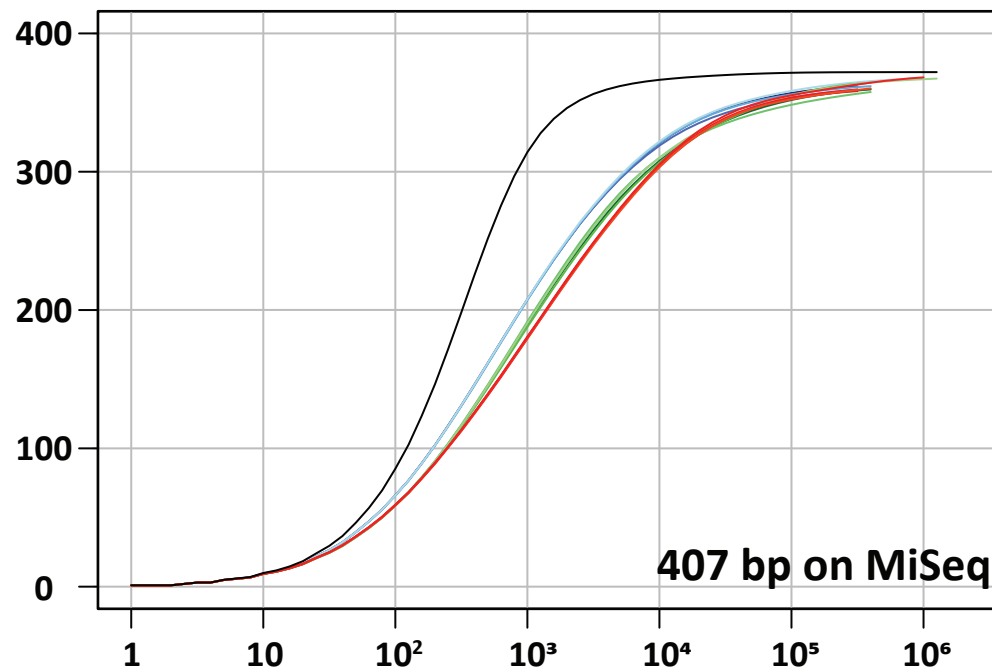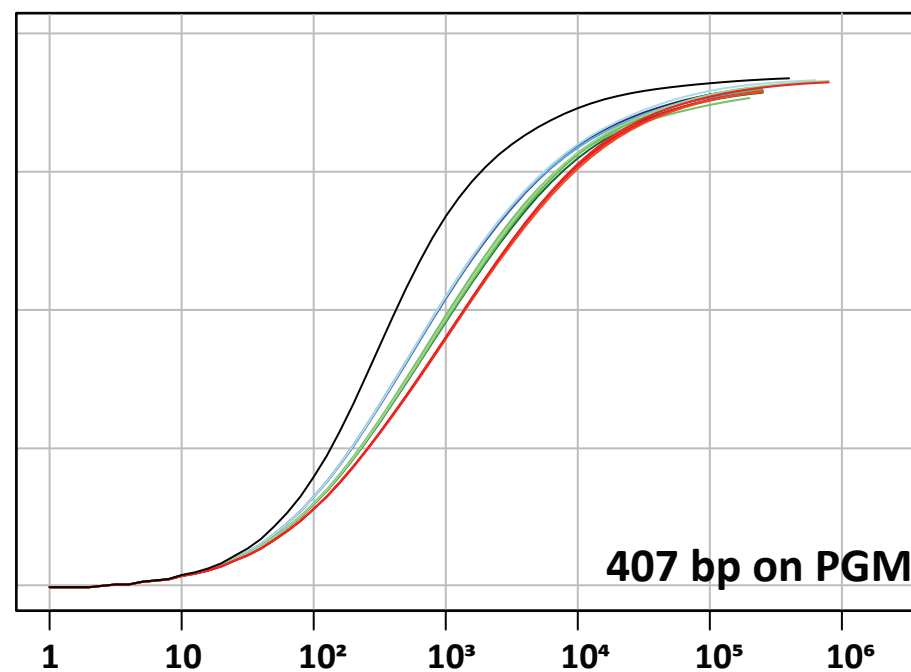

**read depth**

Supplement: Supplementary file 1 [file MEN-19-711-s001.zip › men13008-sup-0002-FigS2.pdf]

diversity

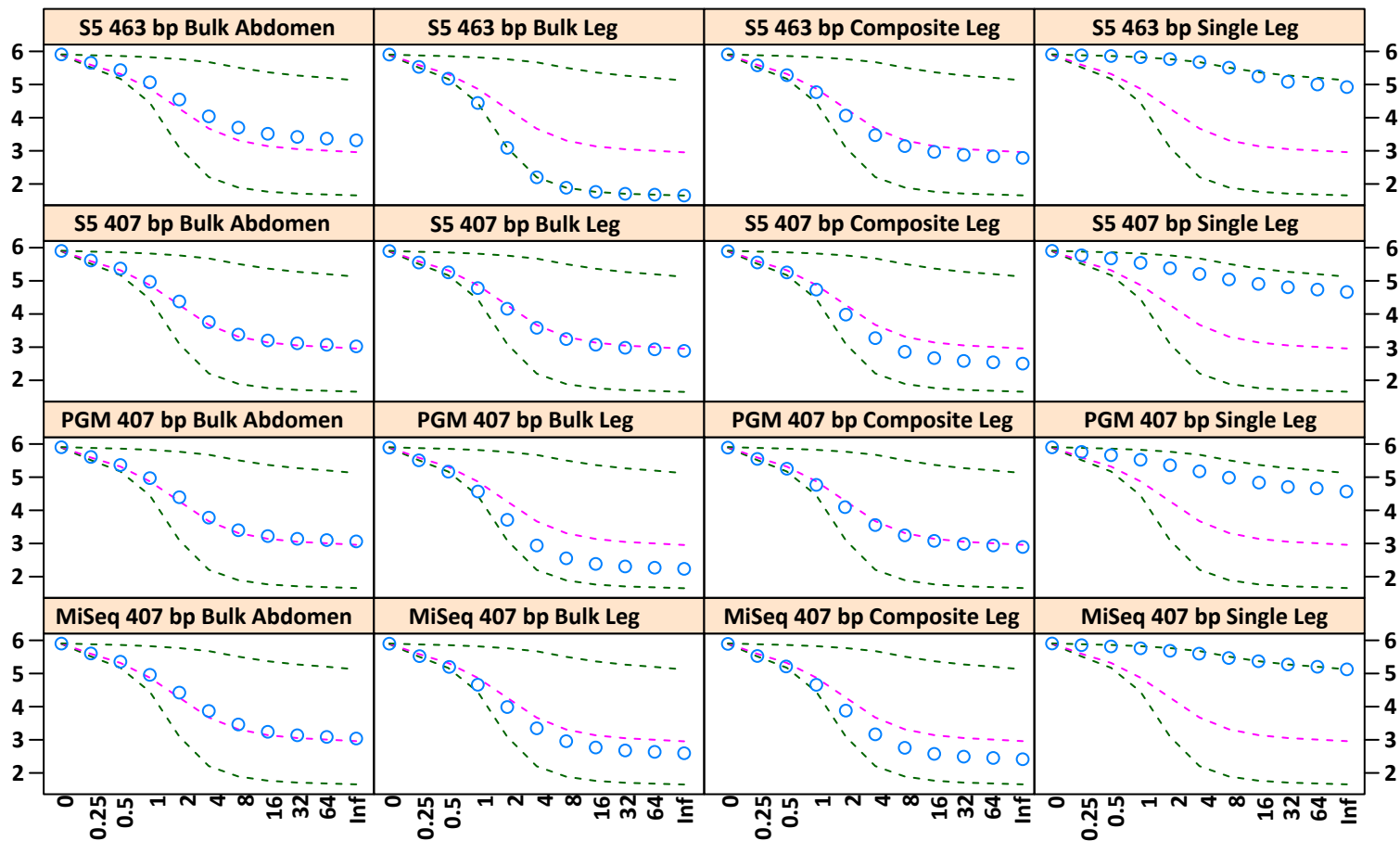

Supplement: Supplementary file 1 [file MEN-19-711-s001.zip › men13008-sup-0003-FigS3.pdf]

Density

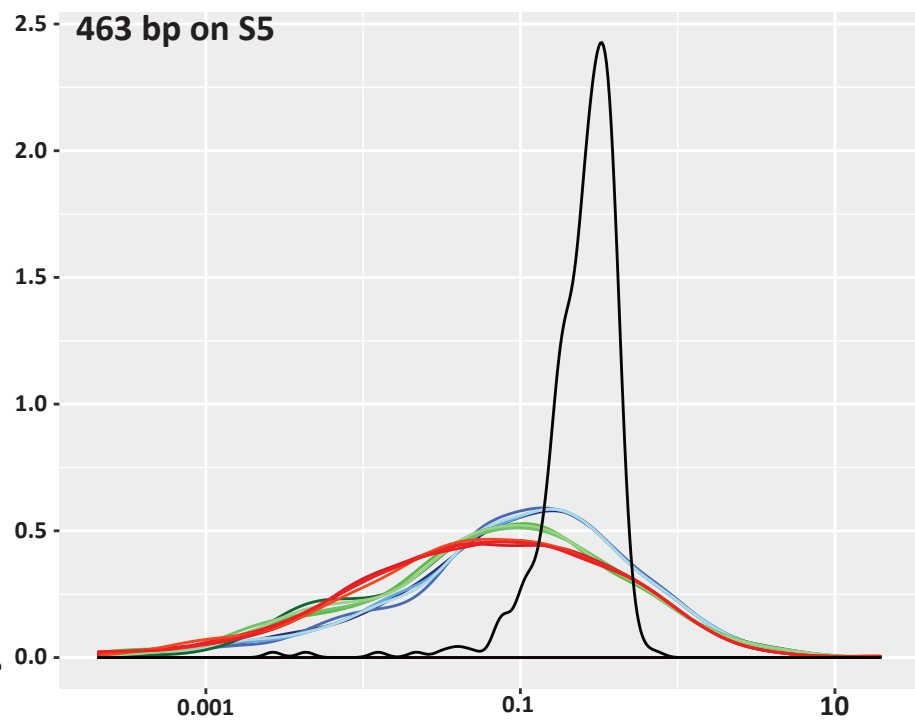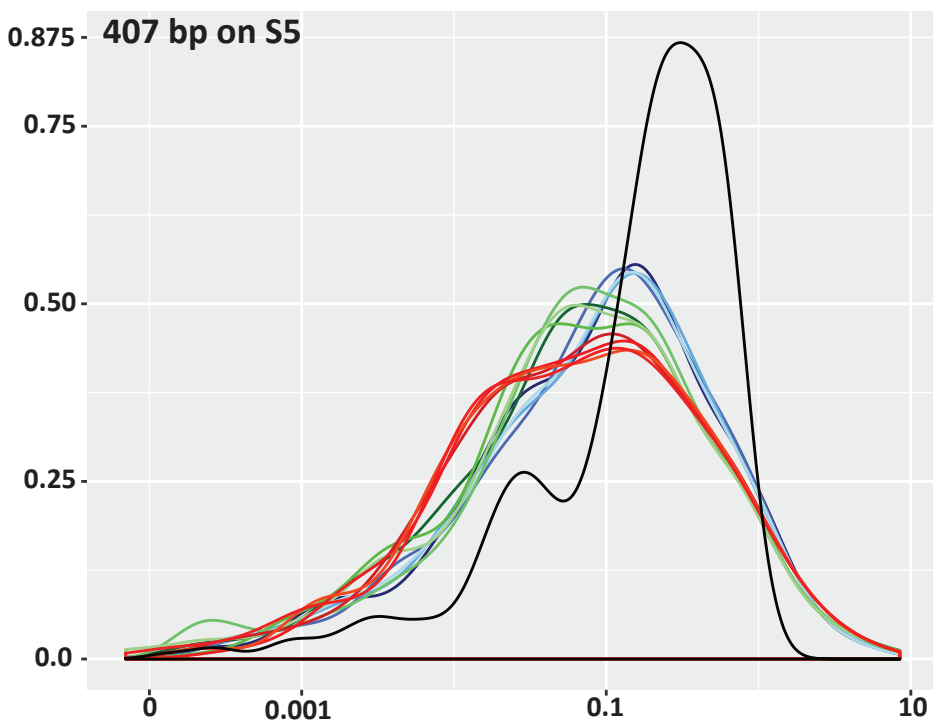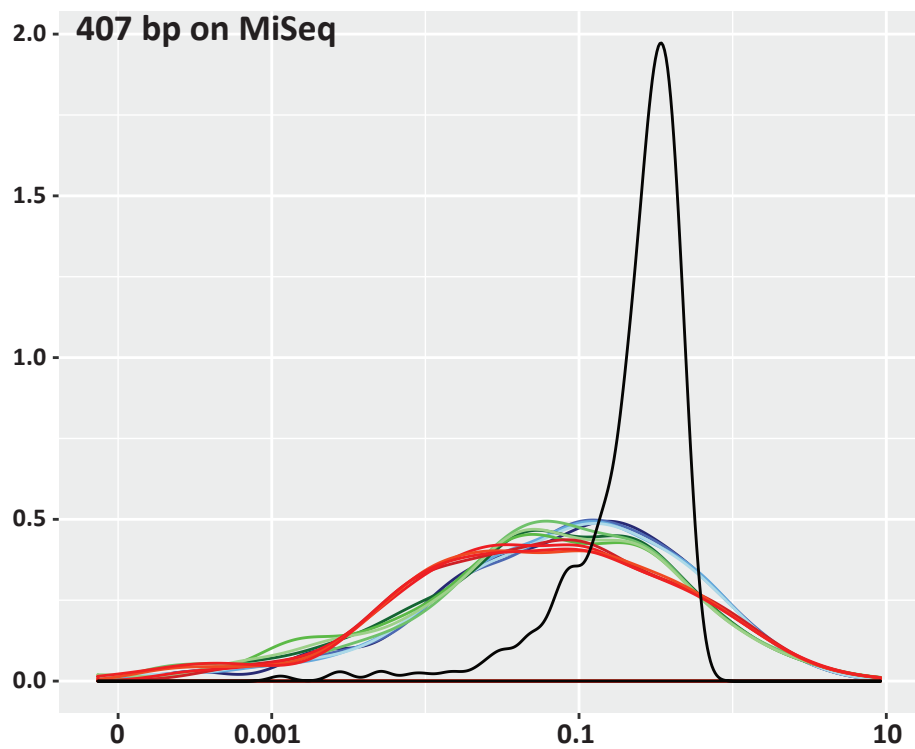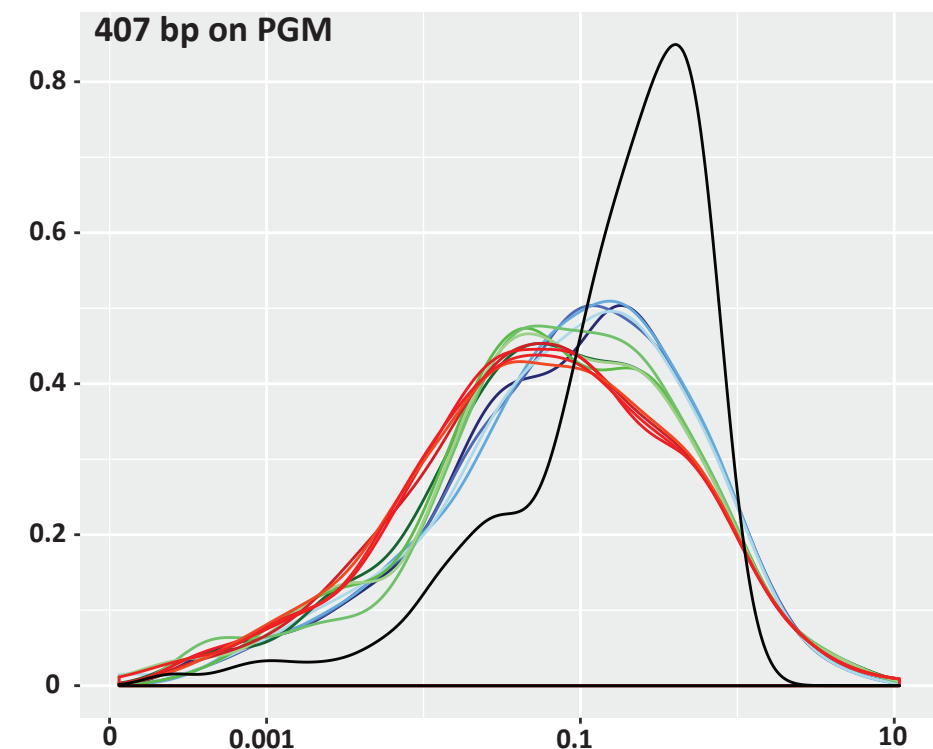

Relative Abundance (%)

Supplement: Supplementary file 1 [file MEN-19-711-s001.zip › men13008-sup-0004-FigS4.pdf]

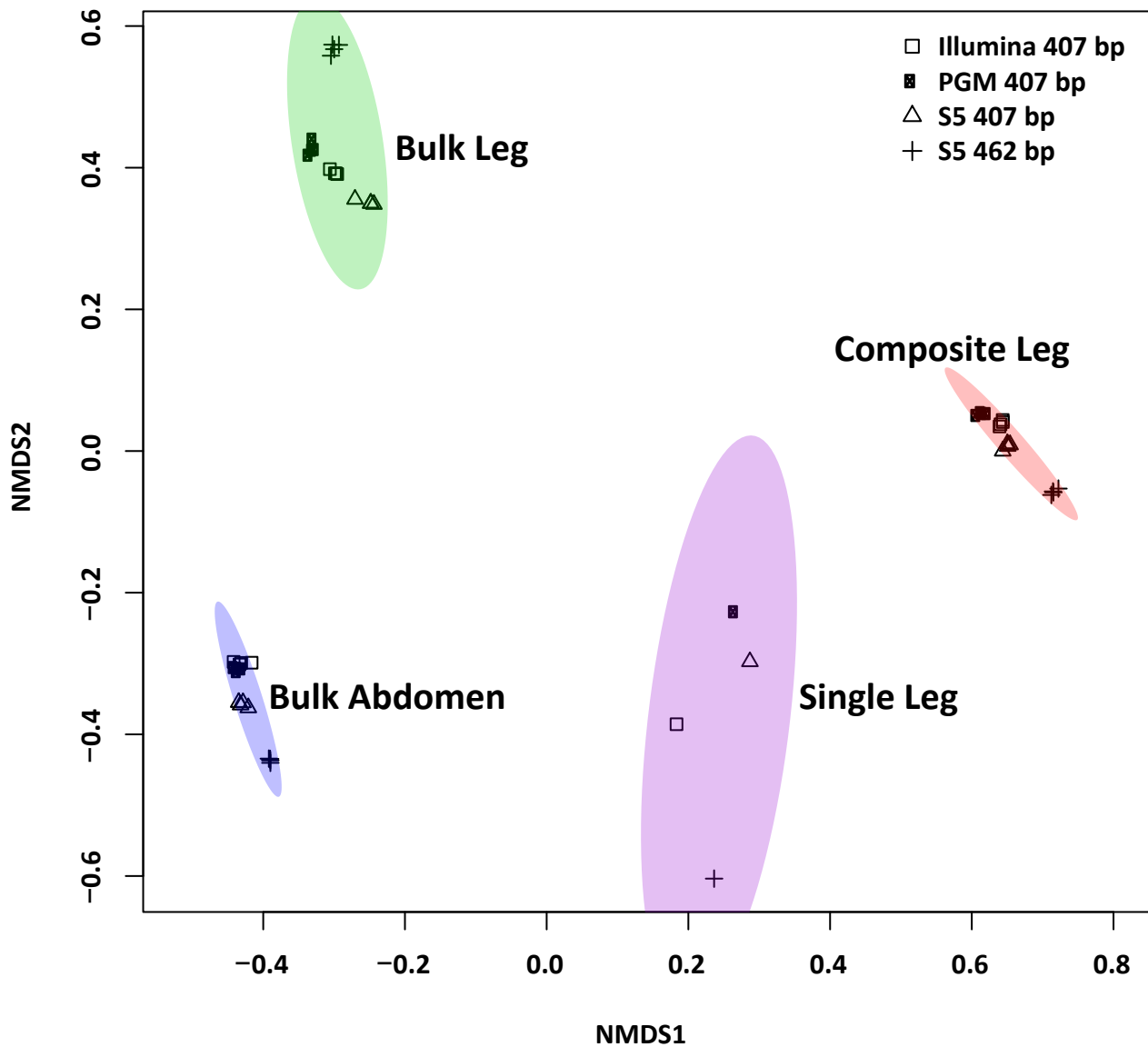

Supplement: Supplementary file 1 [file MEN-19-711-s001.zip › men13008-sup-0005-FigS5.pdf]

relative abundance

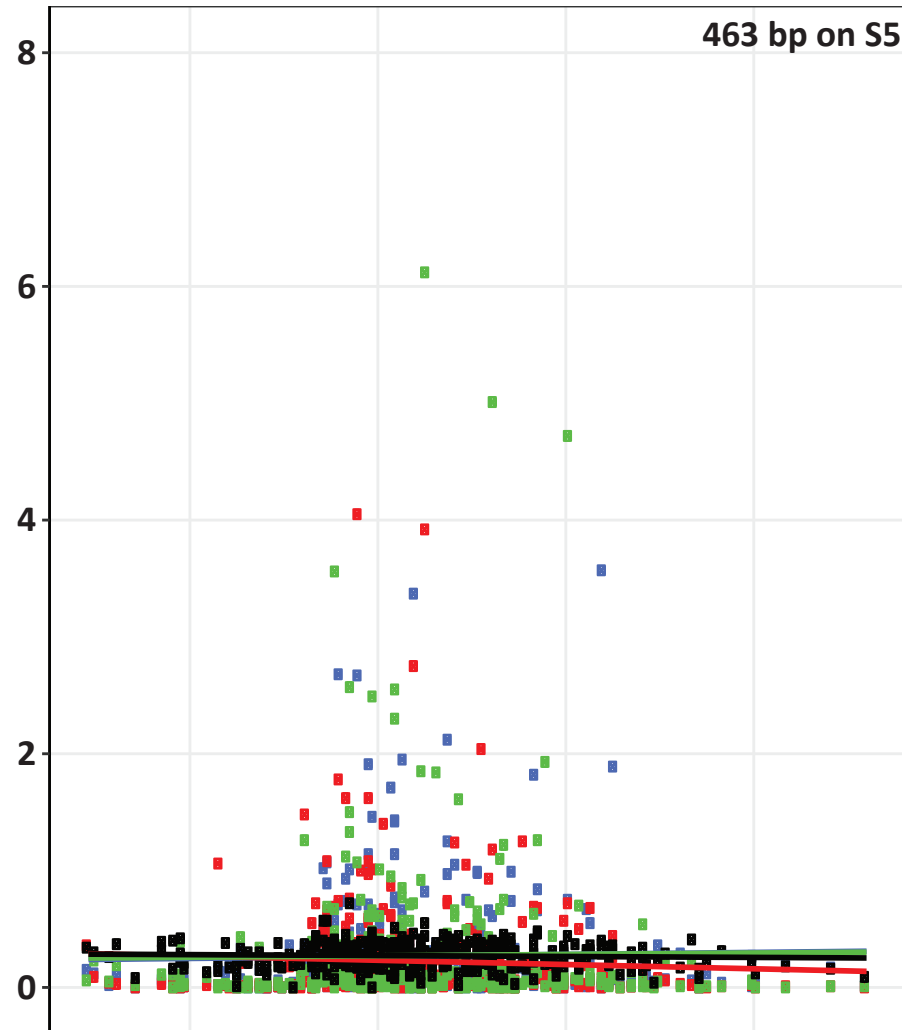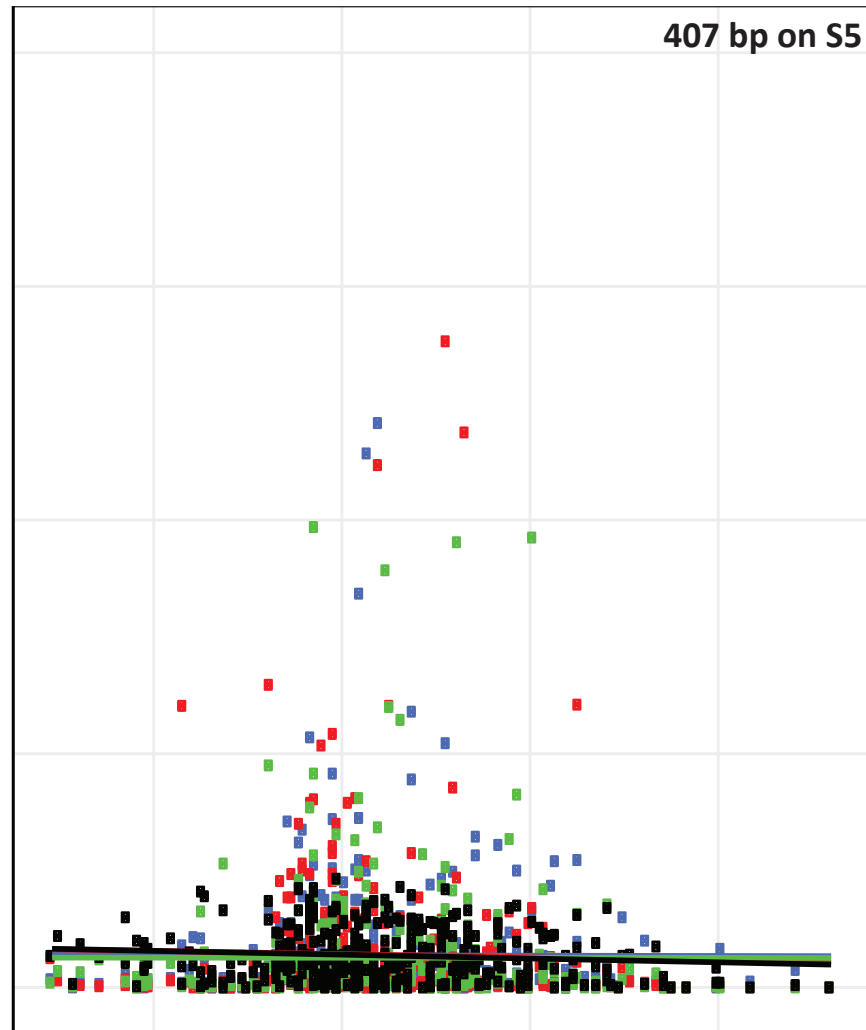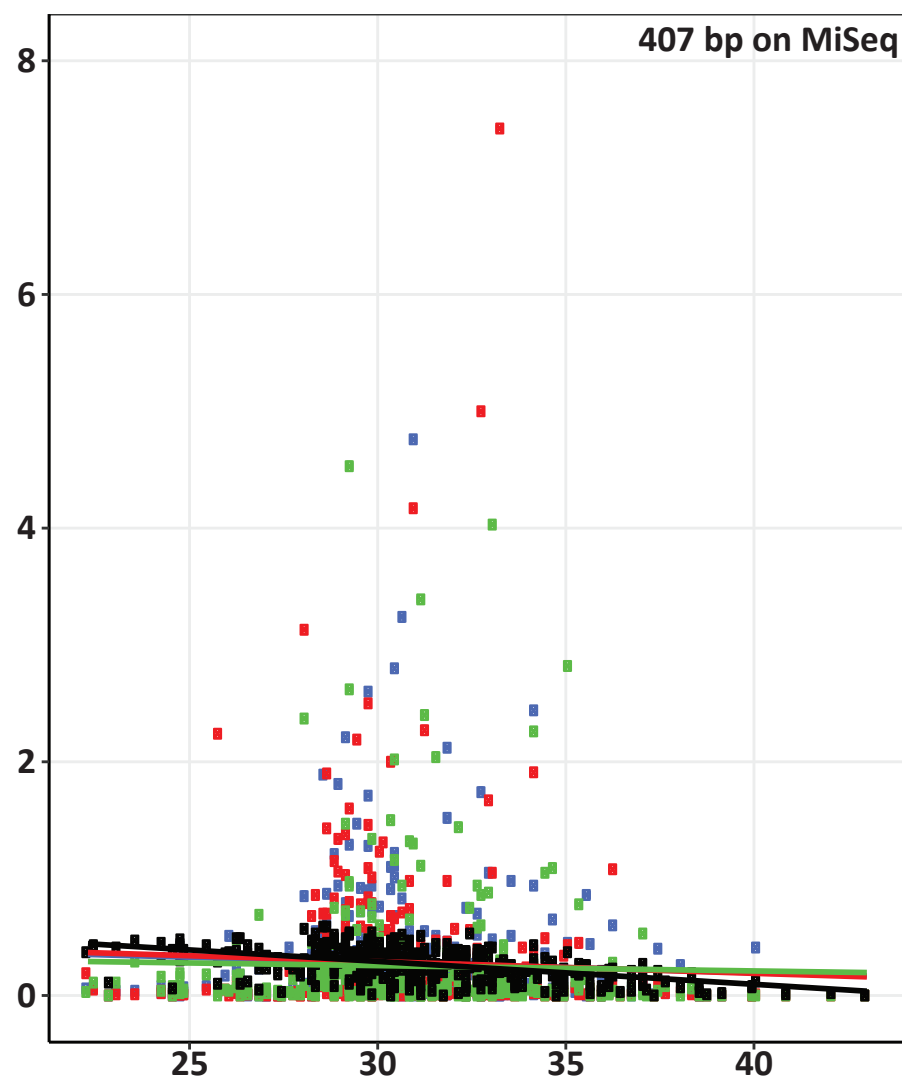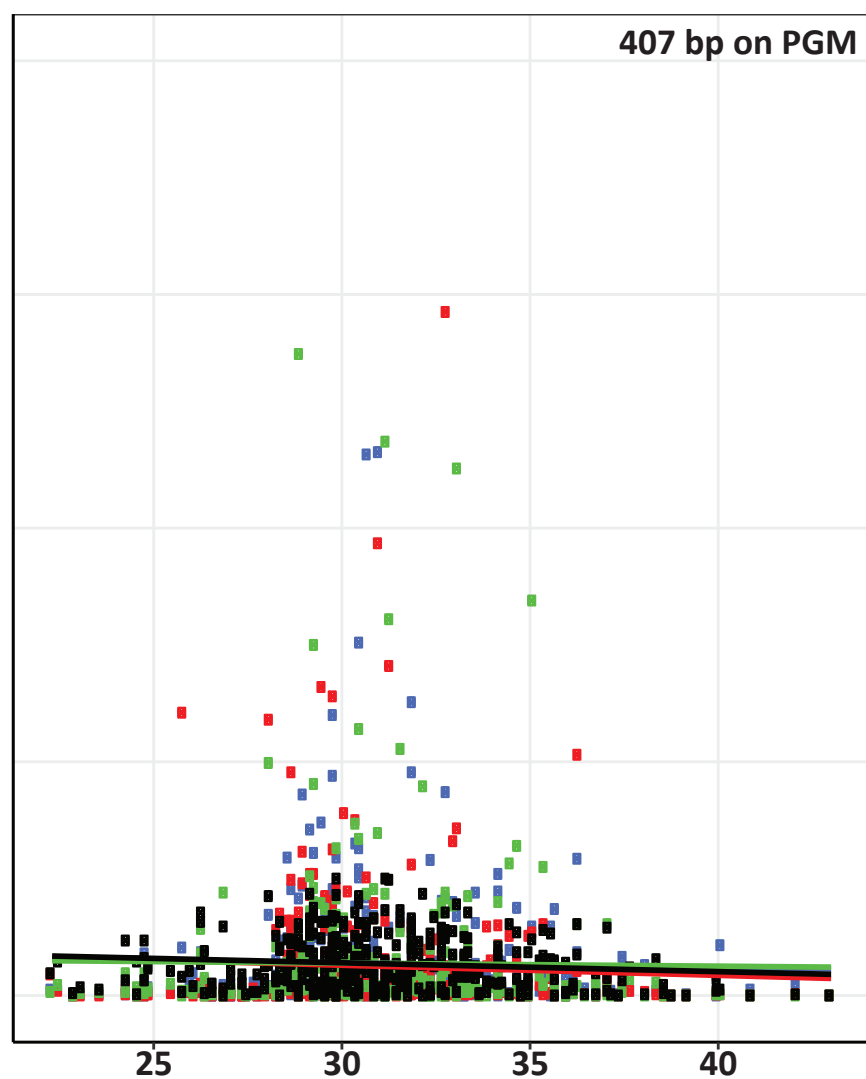

GC (%)

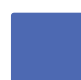

Bulk Abdomen

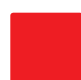

Bulk Leg

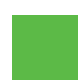

Composite Leg

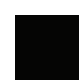

Single Leg

Supplement: Supplementary file 1 [file MEN-19-711-s001.zip › men13008-sup-0007-FigS7.pdf]
