# Supplementary material for: Metabarcoding a diverse arthropod mock community
Source: Mol Ecol Resour. 2019 Apr 20;19(3):711–27. doi: 10.1111/1755-0998.13008 (PMC6850013; doi:10.1111/1755-0998.13008)
Supplement: Supplementary file 1 [file men13008-sup-0006-FigS6.pdf › men13008-sup-0006-FigS6.pdf]

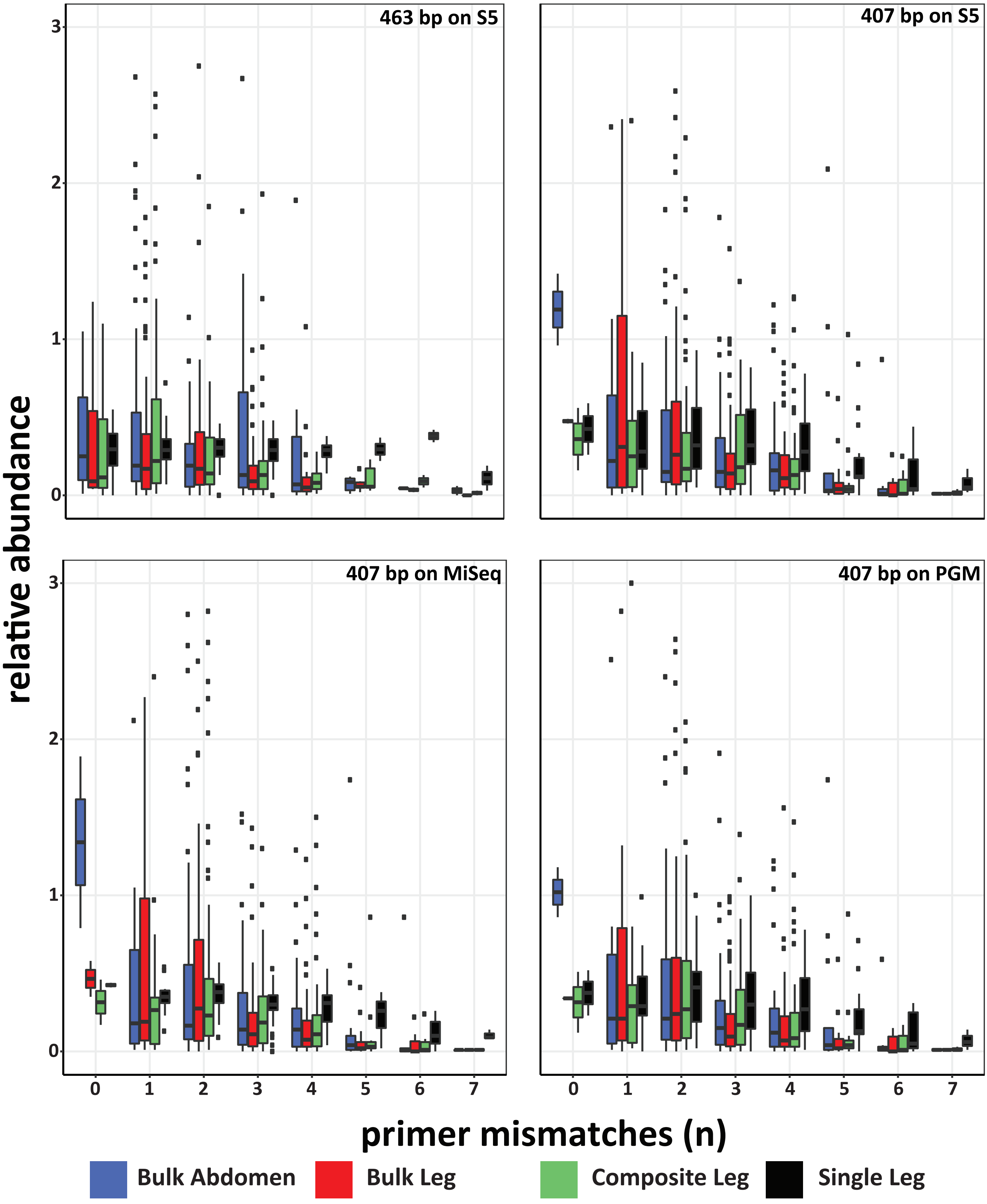

463 bp on S5
407 bp on S5
407 bp on MiSeq
407 bp on PGM
relative abundance
primer mismatches (n)
0
1
2
3
4
5
6
7
Bulk Abdomen
Bulk Leg
Composite Leg
Single Leg
